# Hawthorne Effect with Transient Behavioral and Biochemical Changes in a Randomized Controlled Sleep Extension Trial of Chronically Short-Sleeping Obese Adults: Implications for the Design and Interpretation of Clinical Studies

**DOI:** 10.1371/journal.pone.0104176

**Published:** 2014-08-20

**Authors:** Giovanni Cizza, Paolo Piaggi, Kristina I. Rother, Gyorgy Csako, for the Sleep Extension Study Group

**Affiliations:** 1 Section on Endocrinology & Genetics, Program on Developmental Endocrinology & Genetics, Eunice Kennedy Shriver National Institute of Child Health and Human Development, Bethesda, Maryland, United States of America; 2 Obesity Research Center, Endocrinology Unit, University Hospital of Pisa, Pisa, Italy; 3 Section on Pediatric Diabetes and Metabolism, Diabetes, Endocrinology, and Obesity Branch/National Institute of Diabetes & Digestive & Kidney Diseases, Bethesda, Maryland, United States of America; 4 Department of Laboratory Medicine, Clinical Center, National Institutes of Health, Bethesda, Maryland, United States of America; Charité - Universitätsmedizin Berlin, Germany

## Abstract

**Objective:**

To evaluate the effects of study participation *per se* at the beginning of a sleep extension trial between screening, randomization, and the run-in visit.

**Design:**

Subjects were screened, returned for randomization (Comparison *vs.* Intervention) after 81 days (median), and attended run-in visit 121 days later.

**Setting:**

Outpatient.

**Patients:**

Obese (N = 125; M/F, 30/95; Blacks/Whites/Other, N = 73/44/8), mean weight 107.6±19.7 kg, <6.5 h sleep/night.

**Intervention:**

Non-pharmacological sleep extension.

**Measurements:**

Sleep duration (diaries and actigraphy watch), sleep quality (Pittsburgh Sleep Quality Index), daily sleepiness (Epworth Sleepiness Scale), fasting glucose, insulin and lipids.

**Results:**

Prior to any intervention, marked improvements occurred between screening and randomization. Sleep duration increased (diaries: 357.4 ±51.2 *vs.* 388.1±48.6 min/night; mean±SD; P<0.001 screening vs. randomization; actigraphy: 344.3 ±41.9 *vs.* 358.6±48.2 min/night; P<0.001) sleep quality improved (9.1±3.2 *vs.* 8.2±3.0 PSQI score; P<0.001), sleepiness tended to improve (8.9±4.6 *vs.* 8.3±4.5 ESS score; P = 0.06), insulin resistance decreased (0.327±0.038 *vs.* 0.351±0.045; Quicki index; P<0.001), and lipids improved, except for HDL-C. Abnormal fasting glucose (25% *vs.* 11%; P = 0.007), and metabolic syndrome (42% *vs.* 29%; P = 0.007) both decreased. In absence of intervention, the earlier metabolic improvements disappeared at the run-in visit.

**Limitations:**

Relatively small sample size.

**Conclusions:**

Improvements in biochemical and behavioral parameters between screening and randomization changed the “true” study baseline, thereby potentially affecting outcome. While regression to the mean and placebo effect were considered, these findings are most consistent with the “Hawthorne effect”, according to which behavior measured in the setting of an experimental study changes in response to the attention received from study investigators. This is the first time that biochemical changes were documented with respect to the Hawthorne effect. The findings have implications for the design and conduct of clinical research.

**Trial Registration:**

ClinicalTrials.gov NCT00261898.

## Introduction

Since the first prospective report in 2004 of an inverse association between short sleep and body mass index (BMI) in a cohort of 496 young adults followed for 13 years [1], a growing body of evidence has accumulated on the negative consequences of sleep deprivation on weight, metabolism, and the endocrine system. Chronic sleep deprivation [2] and social jet lag, the modern tendency of living a lifestyle in dissonance with the inherent biological clock [3], have been listed among the emerging factors contributing to the modern obesogenic environment in industrialized societies. A meta-analysis of 45 studies with a total of more than 600,000 children and adults reported an increased risk of obesity in subjects with short sleep duration (odds ratio: 1.55, adults; 1.89, children) [4]. The existence of an inverse relationship between short sleep and BMI is undisputed [5] especially in obese subjects [6], but it remains to be established whether extending sleep would result in weight loss. Prior circumstantial evidence that extending sleep duration and improving sleep hygiene may lead to weight loss has recently been complemented by additional reports providing more direct evidence for a relationship between sleep and weight. A small, controlled, randomized, cross-over study conducted in overweight (average BMI 27 kg/m^2^) men and women showed that moderate (5.5 h vs. 8.5 h) sleep deprivation limits diet-induced fat loss by approximately 50% or 0.7 kg and is accompanied by a shift in relative substrate oxidation toward oxidation of less fat [7]. Furthermore, a 6 year, large, longitudinal study found that subjects sleeping on average less than 6 h/night gained over time approximately 60% more visceral fat than subjects sleeping more than 9 h/night [8].

After bariatric surgery, sleep quality improves substantially and sleep duration increases by approximately 50 min [9] and better sleep quality is associated with higher chances of success in weight loss programs [10] specifically with more fat loss [11].

The existence of a causal relationship is best tested in randomized, controlled clinical trials. Nevertheless, performing randomized trials of behavioral intervention, presents unique challenges. It is difficult to mask subjects to treatment allocation, compliance with study requirements is problematic and requires a high level of participation, and in a randomized study subjects may behave in dissonance with group allocation. In the case of sleep extension subjects in the control group may also decide to extend sleep duration and *vice versa*.

The Sleep Extension Study is the first randomized, controlled trial of sleep extension in chronically sleep-deprived (less than 6.5 h per night) obese subjects. The study design has been previously reported [12]. The main study hypothesis was that sleep extension would cause weight loss and induce metabolic and endocrine improvements. Since the potential impact of the interactions between participants and study team on sleep behavior and various biochemical parameters in the early phases of a behavioral study of sleep extension have not been fully characterized, we investigated it in the above chronically sleep-deprived obese population. In a period of about 2.7 months between screening and randomization (*i.e.*, prior to any intervention), we observed substantial improvements in sleep and select biochemical parameters, most prominently in glucose homeostasis. The improvements were transient and most of them disappeared in the absence of intervention about four months after screening. We propose that the observed changes were due to the Hawthorne effect.

## Methods

The protocol for this trial and supporting CONSORT checklist are available as supporting information; see Checklist S1 and Protocol S1.

### Ethics Statement

The National Institutes of Health (NIH) Institutional Review Board approved the protocol and each individual gave written informed consent for this Sleep Extension Study. The study was conducted at the NIH Clinical Center (CC) according to the principles of the Declaration of Helsinki (ClinicalTrials.gov identifier NCT00261898).

### Study Subjects and Recruitment

Subjects were men and premenopausal women aged 18 to 50 years of age with a body mass index (BMI) between 30 and 55 kg/m^2^ who reported sleeping less than 6.5 h per night. They self-identified their ethnicity as “black”, “white”, or “other”. Recruitment occurred between January 2007 and June 2011 by advertising for obese subjects who reported sleeping less than 6.5 h per night. In addition, body weight had to have remained within 5% over the previous 6 months. The NIH patient recruitment center conducted the initial prescreening phone interview to identify potential candidates. The study team then administered a semi-structured telephone interview for further assessing eligibility and potential candidates were invited to appear in person for additional evaluation.

### Study Design

The *Sleep Extension Study* is a randomized, controlled study of sleep extension in chronically sleep-deprived obese individuals. Additional details have been published [13], [14]. The study was comprised of two different phases, the Efficacy Phase followed by the Effectiveness Phase.

The Efficacy Phase was designed to test whether it was possible to extend sleep duration and to improve sleep hygiene and to characterize the beneficial effects (or lack thereof) of the intervention on weight, endocrine, and metabolic parameters. This phase was conducted as a randomized, parallel group, clinical trial. The study hypothesis was stated in a neutral way (to test whether “changes in sleep duration are paralleled by changes in body weight).

The subsequent Effectiveness Phase was designed to test the feasibility of sleep extension in a more naturalistic setting reminiscent of clinical practice, thus characterized by less frequent coaching and monitoring by the study team. In the Effectiveness Phase every participant, whether previously randomized for the Efficacy Phase to the Intervention or to the Comparison Group, would be asked to extend sleep duration.

The different goals of the two phases were paralleled by the different frequency, the *“tempo”*, of the study visits. In the Efficacy Phase, subjects were to be closely monitored by the means of frequent visits, for a total of 11 visits, over a planned time period of 12 months. In the Effectiveness Phases, subjects were to be evaluated less frequently, at 6-months intervals, for a total of planned 3 years. This report focuses on the first three consecutive visits of the Efficacy Phase, the screening visit (Visit 1), the randomization visit (Visit 2), and the run-in visit (Visit 3).

### Screening, Randomization and Run-In Study Visits and Consents

**Figure 1:**
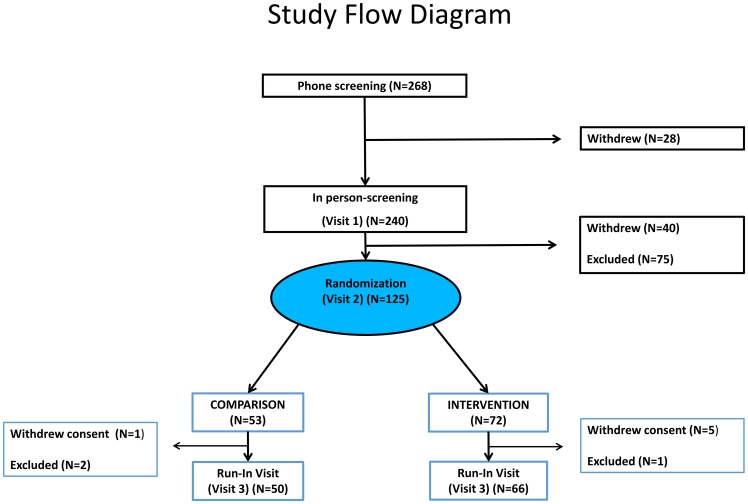
Study flow diagram.

Two hundred and forty individuals were screened in person at the screening visit (Visit 1) (Fig. 1). The main goal of this visit was to ensure that participants met eligibility criteria by eliminating those candidates that had social, medical, or other reasons that could be ascertained in the first in-person encounter with the study team and would prevent enrollment into the active study. The screening consent informed prospective participants that sleep duration and quality would be assessed at each visit, described the devices used, and the procedures involved in the study. This consent also mentioned that eligible participants in the subsequent visit would be assigned by chance to an Intervention or a Comparison Group, should they have qualified for the study. Individuals were asked to wear actigraphy monitors, and complete sleep questionnaires and diaries for two consecutive weeks in a month.

At Visit 2 (randomization visit), 125 of the initially screened 240 individuals were randomized in a 1.3∶1 ratio (Intervention/Comparison), thus assigning 72 to the Intervention Group and 53 to the Comparison Group (Fig. 1). The main goal of this visit was to ensure that participants would be able to comply with the various requirements that the study entailed, based on group allocation. Eligibility was determined based on several parameters of sleep duration such as actigraphy, questionnaires, and diaries. These results were shared with the candidates and those eligible were consented for randomization. The requirements of study participation were described in details in the randomization consent which mentioned that randomization to group assignment would be performed by a computer, based on a specifically designed algorithm. Once the subject was randomized, group assignment was disclosed and the subject was asked to sign another consent specific for the Intervention or for the Comparison Group, depending on the results of the randomization. Subjects in the Intervention Group were instructed to increase sleep duration up to 7.5 h per night, following a personalized sleep plan devised in close collaboration with the subject and based on the information collected up to that point. Strategies included consistent bedtime routine, avoiding caffeine, alcohol, heavy meals and exercise prior to bedtime, creating an environment conducive to sleep, and controlling bedroom light and temperature. The Comparison Group was asked not to modify the existing short sleep habits for the remaining time of the Efficacy Phase of the study. The rest of the two consents were identical. At the Run-In Visit, the sleep results between Visit 2 and Visit 3 were discussed with each participant.

Of all participants randomized, a total of 116 subjects made it to the run-in visit (Visit 3): 50 in the Comparison Group and 66 in the Intervention Group (Fig. 1). The goal of this visit was to verify the level of compliance with the group-specific instructions received at Visit 2, namely to increase their sleep duration (Intervention Group) or to maintain their sleep duration (Comparison Group). In addition, subjects in both groups were required to record a two-week diary of sleep time and were asked not to attempt to lose weight.

### Study Procedures

#### Anthropometric measurements

Standing height was measured using a wall-mounted stadiometer (SECA 242, SECA North America East, Hanover, MD) to the nearest millimeter three times and the mean of these measurements was determined and weight was determined using a stand-on scale in a hospital gown to the nearest 1/10th of a kg (SR555 SR Scales, SR Instruments, INC, Tonawanda, NY). Waist circumference (WC) was determined by placing the measuring tape in a horizontal plane around the abdomen above the uppermost lateral border of the right iliac crest at the end of a normal expiration. If this site could not be determined, the maximum circumference was observed at or near the level of the umbilicus. Neck circumference (NC) was measured at the minimal circumference with the subjects' head in the Frankfurt Horizontal Plane. Metabolic syndrome was defined according to the ATP-III criteria [15]. Subjects were given an electronic scale that they could keep at the end of the study and asked to record their weight on a weekly basis; they were also provided with a blood pressure instrument and instructed to measure and record their blood pressure on a weekly basis in conjunction with the weight measurement.

#### Sleep parameters

Sleep duration was estimated by sleep diaries and by wrist actigraphy (Actiwatch-64) worn continuously for 2 weeks (average 13±2 days, median 13, range 5–15). Two measures were included: sleep duration (amount of nighttime sleep obtained during 24 h), and sleep efficiency (percentage of time in bed spent sleeping). Participants completed the Pittsburgh Sleep Quality Index (PSQI), a validated 21-item questionnaire with inquiries about sleep, including quality over the past month. Scores were dichotomized at ≤5 or >5, the conventional threshold for poor sleep quality [16]. Daytime sleepiness was assessed by the Epworth Sleepiness Scale (ESS), a validated 8-item questionnaire [17]. Higher scores represent increased daytime sleepiness; values >10 indicate abnormal sleepiness. Participants received instructions for maintaining sleep diaries for the following two weeks, and for wearing an actigraphy monitor (Actiwatch-64, Mini Mitter/Respironics/Philips, Bend, OR) over that same period of time.

#### Clinical laboratory analysis

Morning fasting venous blood was obtained for laboratory analyses. Plasma glucose and serum triglycerides, total cholesterol, low-density lipoprotein cholesterol (LDL-C) and high-density lipoprotein cholesterol (HDL-C) were measured on a Dimension Vista 1500 analyzer (Siemens Health Diagnostics, Deerfield, IL), whereas serum insulin was measured with a chemiluminescence immunoassay (Immulite 2000, Siemens).

### Statistical Analysis

**Figure 2:**
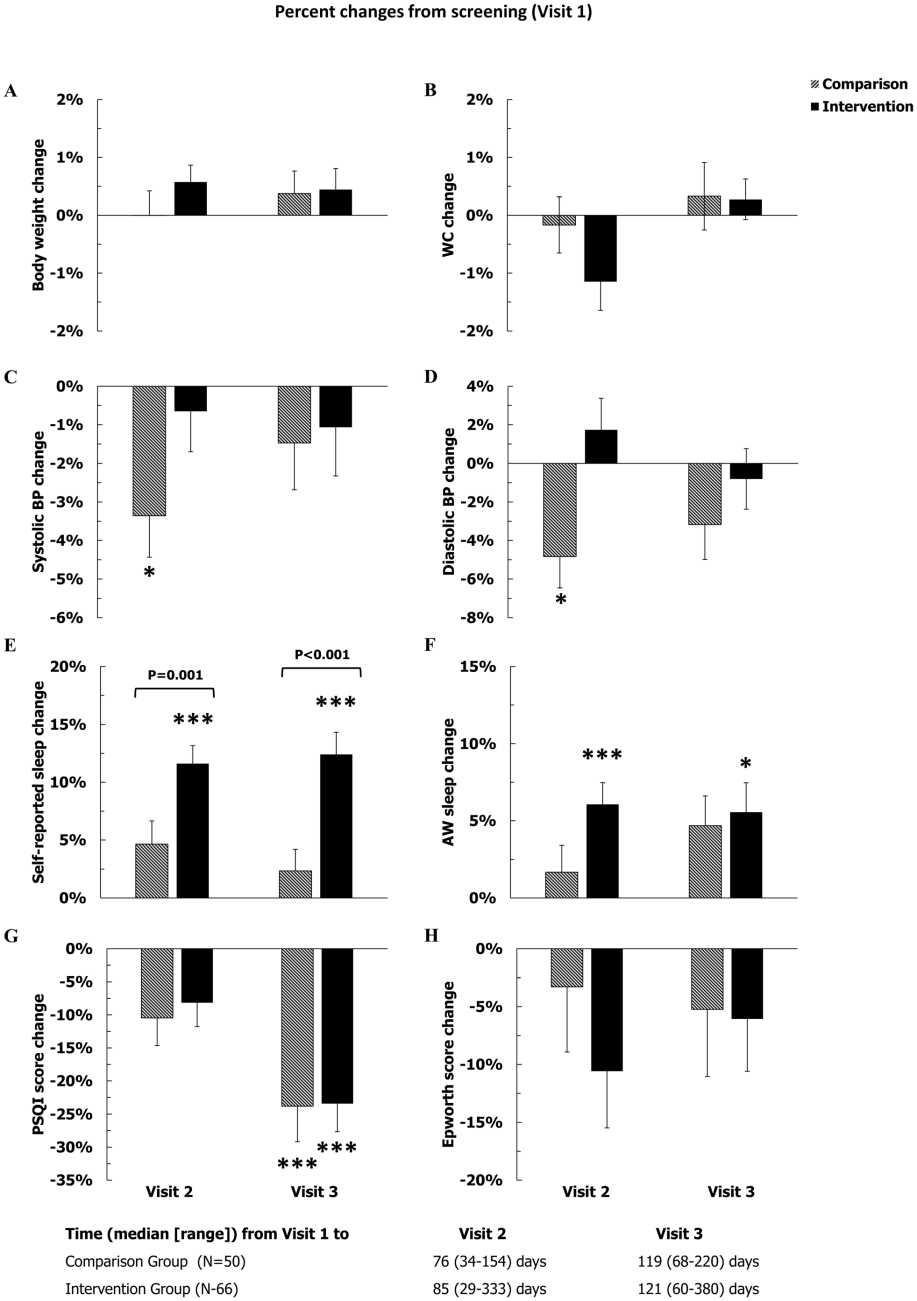
Percent changes from screening in weight and waist circumference (WC), and in systolic and diastolic blood pressure (upper four panels, A–D), and the changes in sleep duration by diaries and by actigraphy watch (AW), and the changes in PSQI, and Epworth scores (lower four panels, E–H). Error bars represent 1 SEM. Significant changes from screening: *P<0.05, **P<0.01, and ***P<0.001; all adjusted for multiple testing. Significant between group differences (Comparison vs. Intervention) are shown with actual P-value, as appropriate, above or below the pair of columns. The time intervals between Visit 1 and Visit 2, as well as Visit 1 and Visit 3 were not different between the two groups (P = 0.30 and P = 0.36, respectively).

**Figure 3:**
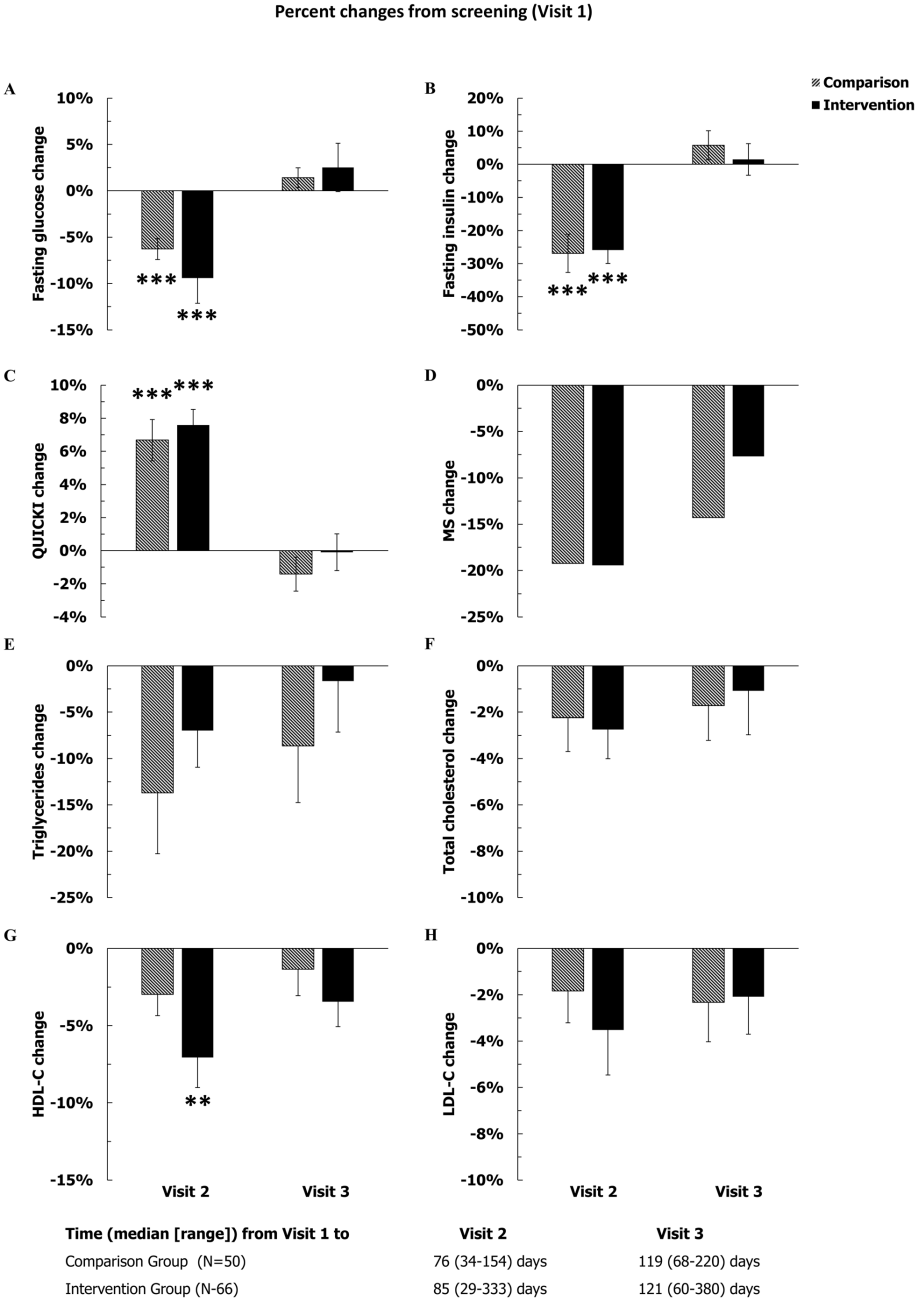
Percent changes from screening in glucose parameters and in the prevalence of metabolic syndrome (upper four panels, A–D) and the changes in lipid levels (lower four panels, E–H). Error bars represent 1 SEM. Significant changes from screening: *P<0.05, **P<0.01, and ***P<0.001; all adjusted for multiple testing. Significant between group differences (Comparison vs. Intervention) are shown with actual P-value, as appropriate, above or below the pair of columns. The time intervals between Visit 1 and Visit 2, as well as Visit 1 and Visit 3 were not different between the two groups (P = 0.30 and P = 0.36, respectively).

The Kolmogorov-Smirnov test was used to assess normality of data; logarithmic transformations were employed for skewed variables (e.g., insulin concentrations) before parametric tests were performed. Statistical tests used to compare pairwise changes between visists included paired Student t-test for difference in mean values, Wilcoxon test for skewed variables and, as appropriate, Chi square or McNemar test for difference in counts and frequency. Mixed models analysis accounting for repeated measures and missing data was used to assess changes between groups and within each group over Visits 1–3. Post-hoc tests were conducted using the Bonferroni correction of significance. P-values were also adjusted for multiple testing according to the independent classes of parameters (n = 4), namely, sleeping, anthropometric, glycemic and lipid characteristics. Analyses were performed using SPSS (version 21, IBM Corp, Armonk, NY, USA). Data are presented as mean±standard deviation (SD) in the tables and standard error of the mean (SEM) in the error bars of Figs. 2 and 3.

## Results

### Characterization of the Participants at Screening (Visit 1) in the Full Study Population

Of the 240 individuals screened in person at Visit 1, 125 participants completed Visit 2 (thereby referred to as “full study population”) and 116 completed all three visits. Mean age was 41 years, mean BMI 38 kg/m^2^, approximately 75% were women, and more than half were black at the time of screening (Table 1). Average sleep duration was less than 6 h: 357 min by diaries, and 344 min by actigraphy. Sleep efficiency was 80% and the average score for ESS and PSQI questionnaires was 9. Thus, sleep parameters reflected inadequate sleep, both in quantity and quality at the time of screening.

**Table 1:** Characteristics of Participants at Screening (Visit 1) and Randomization (Visit 2).

	Screening (Visit 1) (N = 125)	Randomization (Visit 2) (N = 125)	P-Value
*Demographic characteristics*			
Age (yrs)	40.6±6.9	na	na
Male sex (no. (%))	30 (24%)	na	na
Black	73 (59%)	na	na
White	44 (35%)	na	na
Other	8 (6%)	na	na
*Sleep characteristics*			
Sleep duration by diary (min/night)	357.4±51.2 (N = 91)	388.1±48.6 (N = 91)	**<0.001**
Sleep duration by actigraphy (min/night)	344.3±41.9 (N = 118)	358.6±48.2 (N = 118)	**<0.001**
Sleep efficiency (%)	79.5±8.0 (N = 114)	79.9±6.5; 0.5 (N = 114)	0.645
Subjects with abnormal sleep efficiency N (%) (less than 80%)	52 (42%)	47 (38%)	0.601
1Daytime sleepiness (ESS score)	8.9±4.6 (N = 117)	8.3±4.5 (N = 117)	0.055
Subjects with abnormal ESS score N (%) >5	87 (70%)	85 (68%)	0.837
2Subjective sleep quality (PSQI score)	9.1±3.2 (N = 112)	8.2±3.0 (N = 112)	**0.001**
Subjects with abnormal PSQI score N (%) >10	37 (30%)	26 (21)	0.573
*Anthropometric parameters and blood pressure*			
BMI (kg/m^2^)	38.4±6.1	38.5±6.3	0.232
Weight (kg)	107.6±19.7	108.0±20.4	0.169
Waist circumference (cm)	114.6±13.3	113.8±13.1	**0.038**
Neck circumference (cm)	39.2±3.9	39.2±3.9	0.303
Systolic Blood Pressure (BP) (mm/Hg)	126.5±12.3	124.3±12.2	**0.020**
Subjects with abnormal systolic BP (>120 mmHg)	86 (69%)	77 (62%)	0.302
Diastolic BP (mm/Hg)	75.1±9.7	74.2±9.8	0.365
Subjects with abnormal diastolic BP N (%) (>80 mmHg)	36 (29%)	37 (30%)	0.972
*Biochemical parameters*			
Fasting glucose (mg/dL)	97.7±17.6 (N = 123)	89.7±12.6 (N = 123)	**<0.001**
Subjects with abnormal fasting glucose N (%) >100 mg/dL	31 (25%)	14 (11%)	**0.007**
Fasting Insulin (µIU/mL)	15.5±8.7 (N = 124)	11.4±8.6 (N = 124)	**<0.001**
Quicki Index	0.327±0.038 (N = 122)	0.351±0.045 (N = 122)	**<0.001**
Triglycerides (mg/dL)	108.7±75.1	97.9±53.5	**0.007**
Subjects with abnormal triglycerides N (%) >150 mg/dL	23 (18%)	19 (15%)	**0.639**
Total cholesterol (mg/dL)	184.1±36.9	179.4±35.5	**0.009**
Subjects with abnormal total cholesterol N (%) >200 mg/dL	35 (28%)	27 (22%)	0.342
HDL-C (mg/dL)	52.3±15.0	49.6±13.0	**<0.001**
Proportion of subjects with abnormal HDL-C below 40 mg/dL for men or 50 mg/dL for women	58 (46%)	59 (47%)	0.975
LDL-C (mg/dL)	116.4±32.0	113.1±31.0	**0.037**
Proportion of subjects with abnormal LDL-C >100 mg/dL	81 (65%)	75 (60%)	0.493
Metabolic Syndrome	52 (42%) (N = 124)	36 (29%) (N = 124)	**0.007**

Data is reported as mean±SD or count (frequency).

**: P<0.05.

1 Daytime sleepiness (ESS score): a score of 10 or more is considered sleepy.

2 Subjective sleep quality (PSQI score): a score of 5 or more is considered abnormal.

### Changes Observed between Screening (Visit 1) and Randomization (Visit 2) in the Full Study Population

The median time interval between Visit 1 and Visit 2 was 81 days. During this interval, sleep duration increased significantly by an average of 31 minutes according to diaries, and 14 minutes by actigraphy (Table 1). Sleep quality improved significantly and sleepiness tended to improve as well. On average, waist circumference decreased by 1 cm, systolic blood pressure (SBP) decreased by 2 mmHg, fasting glucose decreased by 9 mg/dL, fasting insulin decreased by 4 IU/mL, and the Quicki index improved by 7%. Also, triglycerides, total cholesterol, and LDL-C were all significantly improved; only HDL became significantly worse. The proportion of subjects with abnormal fasting glucose values decreased significantly from 25% to 11% and the prevalence of metabolic syndrome decreased significantly from 42% to 29%. Despite marked improvements in both sleep and metabolic parameters at Visit 2, there was no significant correlation between their absolute changes (all P>0.05).

### Characterization of the Participants at Screening (Visit 1) According to Subsequent Randomization Status

Since at randomization (Visit 2) participants received different instructions about sleep behavior depending on their group assignment (Intervention *vs.* Comparison), we could not compare results of the “full study population” at the run-in visit (Visit 3) with results at screening and randomization. In order to follow up on possible changes up to Visit 3, we therefore analyzed the percent changes at Visit 2 and Visit 3 compared to Visit 1 according to their “future” group allocation in the 116 participants who reached the run-in phase (Visit 3).

Table 2 reports subjects' characteristics at screening, categorized according to the two allocation groups. Randomization at Visit 2 was successful in achieving comparable characteristics between the Comparison and Intervention Groups for: demographic, sleep and lipid parameters (Table 2). Differences were found in one anthropometric parameter: the waist circumference was 5 cm larger in the Intervention group. This group also tended to have higher body weight and neck circumference, and had significantly higher fasting glucose concentrations and a worse Quicki index.

**Table 2:** Characteristics at screening (Visit 1) of participants who completed randomization.

	Comparison Group (N = 50)	Intervention Group (N = 66)	P-Value
*Demographic characteristics*			
Age (yrs)	41.7±7.3	40.0±6.7	0.201
Male sex (no. (%))	13 (26%)	17 (26%)	0.976
Black	33 (66%)	36 (55%)	0.315
White	14 (28%)	25 (38%)	0.352
Other	3 (6%)	5 (7%)	0.870
*Anthropometric parameters and blood pressure*			
BMI (kg/m^2^)	37.4±5.7	39.0±6.7	0.165
Body weight (kg)	103.4±16.1	109.9±22.3	0.086
Waist circumference (cm)	111.3±11.8	116.4±13	**0.033**
Neck circumference (cm)	38.6±3.8	39.9±4.2	0.086
Systolic blood pressure (BP) (mmHg)	127.5±9.8	126.5±13.1	0.669
Diastolic BP (mmHg)	76.3±9.6	75±9.4	0.442
*Sleep characteristics*			
Sleep duration by diary (min/night)	363.7±49.7	358±53.2	0.586
Sleep duration by actigraphy (min/night)	346.4±43.4	344±41.3	0.761
Sleep efficiency (%)	80.2±6.8	80.2±6.2	0.989
1Daytime sleepiness (ESS score)	9.4±4.6	8.4±4.7	0.277
2Subjective sleep quality (PSQI score)	8.9±3.2	9.1±3.3	0.834
*Biochemical parameters*			
Fasting glucose (mg/dL)	93.8±7.2	101.3±22.8	**0.028**
Fasting Insulin (µIU/mL)	14.5±8.7	17.1±8.7	0.117
Quicki Index	0.334±0.043	0.318±0.032	**0.028**
Triglycerides (mg/dL)	112.6±77.7	109±76.7	0.801
Total cholesterol (mg/dL)	184.6±29.1	182.4±42.3	0.760
HDL-C (mg/dL)	54.7±14.3	50.4±15.1	0.126
LDL-C (mg/dL)	112.7±28.1	117.4±35.1	0.443
Metabolic Syndrome (no. (%))	20 (40%)	29 (44%)	0.671

(Visit 2) and run-in (Visit 3).

Data is reported as mean±SD or count (frequency).

*: P<0.05.

1 Daytime sleepiness (ESS score): a score of 10 or more is considered sleepy.

2 Subjective sleep quality (PSQI score): a score of 5 or more is considered abnormal.

### Percent Changes vs. Screening (Visit 1) at Randomization (Visit 2), and Run-In (Visit 3) in the Comparison and Intervention Groups

The median intervals between Visit 1 and Visit 2 were 76 (IQR: 58–98) and 85 (IQR: 57–112) days in the Comparison and Intervention Group, respectively, (Figs. 2 and 3). Due to the smaller sample sizes, the statistical significance of the changes previously observed in the full population of study subjects (n = 125) between Visit 1 and Visit 2 was not totally reproduced in the two allocation groups ((Comparison (n = 50) and Intervention Groups (n = 66)). In the Comparison Group, the systolic and diastolic blood pressures were significantly lower at Visit 2 than Visit 1, by an average of 3.5% and 4.5%, respectively. In the Intervention Group, self-reported sleep duration and sleep duration by actigraphy were significantly higher than at Visit 1, by 12% and 6% respectively. HDL-C decreased significantly by approximately 6% only in the Intervention Group. On the other hand, even with smaller sample sizes, the improvements in the glucose homeostasis (fasting glucose, fasting insulin, and the Quicki index) were clearly present in both groups (Figs. 2 and 3).

The median intervals between Visit 2 and Visit 3 were 35 (IQR: 30–42) and 35 (IQR: 28–49) days in the Comparison and Intervention Groups, respectively. In the Intervention Group, as expected from the study intervention, several sleep parameters improved significantly by Visit 3 compared to Visit 1 (Fig. 2): self-reported sleep duration, sleep duration by actigraphy, and sleep quality all improved by an average of 13%, 5%, and 22% respectively. At Visit 3, sleep quality (Fig. 2, panel G) was also significantly better than at Visit 1 in the Comparison Group. Finally, both at Visit 2 and at Visit 3, the percent increase in self-reported sleep duration was significantly larger in the Intervention Group than in the Comparison Group.

## Discussion

In our Sleep Extension Study of chronically short-sleeping obese adults, we found that sleep duration, sleep quality, and sleepiness, diastolic blood pressure, and insulin sensitivity markedly improved prior to any intervention in about 2.7 months between screening and randomization. The proportion of subjects with pre-diabetes was halved and the proportion of subjects with metabolic syndrome decreased by one third. Since no life-style modifications had been recommended at that point, we were surprised by the magnitude and consistency of the improvements observed after enrollment across a variety of different parameters. Of importance is that several of these parameters were of biochemical nature and therefore measured objectively.

Between screening and randomization, self-reported sleep duration increased by an average of 30 min, which is a clinically meaningful improvement. For comparison, a drug for the treatment of insomnia was recently approved in the US based on a sleep extension of approximately 14 min [18]. The 8% reduction in fasting glucose, paralleled by a 26% decrease in fasting insulin, and by a 7% improvement in Quicki index, an accepted marker of insulin sensitivity, are also remarkable in our subjects. These changes clearly exceeded the “normal” intra-individual fluctuations for most of the parameters measured, which is estimated to be, for example, approximately 4%, and 21% for fasting glucose and fasting insulin, respectively [19]. Further, in the absence of active intervention, glucose parameters tend to get worse over time and certainly do not improve in subjects with the degree of obesity seen in our study. A possible explanation for improvements in glucose homeostasis parameters is the parallel improvement in sleep. Besides temporal sequence, biological plausibility, magnitude and direction of changes and published reports favor this possibility. A large body of evidence indicates that acute sleep deprivation causes insulin resistance in healthy, lean subjects [20]. A meta-analysis of randomized, controlled, one-year duration studies of diet and exercise for patients with metabolic syndrome reported reduced mean values of approximately 11 mg/dL of fasting glucose [21], quite close to the approximately 8 mg/dL reduction in fasting glucose that we observed here. Lipid parameters also showed statistical improvements between screening and randomization; the changes were, however, of smaller magnitude than changes in glucose parameters and insufficient to cause reversal to normal values in a significant proportion of participants. A relationship between sleep and the lipid concentrations has been reported before. Both short and long sleep were associated in women but not in men with abnormal triglycerides, HDL-C, and LDL-C levels in a large survey conducted in Japan of approximately 4000 adult men and women [22]. Nonetheless, in addition to the documented sleep extension, we should also consider the possibility that the marked metabolic changes observed in our subjects between screening and randomization may have been the consequence of unrecognized life-style changes, e.g., those in diet and exercise, as well. Finally, a 1/3 decline in the proportion of our subjects with metabolic syndrome between screening and randomization was driven by the improvements observed in blood pressure, fasting glucose and waist circumference, rather than by improvements in the lipid components of the metabolic syndrome, namely triglycerides and HDL-C.

Due to the study design, follow-up on the changes beyond randomization could only be analyzed in the two groups (Comparison and Intervention Group) of our study population. Once participants were randomized, improvements in sleep parameters were consistently observed only in the group advised to extend sleep at the time of randomization (Intervention Group). Since these subjects have been made fully aware of the goals of the study and have been consented to change their sleep behavior, these effects are expected and they are understood as a direct result of study intervention. On the other hand, irrespective of group allocation at randomization, the initial improvements in parameters of the glucose homeostasis completely disappeared by the time of the Run-In Visit, about 4 months after screening. This was surprising, since this was observed even in the Intervention Group that sustained sleep improvements at the run-in visit.

While we cannot fully explain the specific cause(s) of sleep and biochemical improvements observed between screening and randomization in our study population, we propose that they are consistent with the Hawthorne effect. Of note is that, to the best of our knowledge, no objectively measurable changes have been reported yet as a consequence of the Hawthorne effect. This effect is named after experiments conducted in the 1930s' at the Hawthorne Works plant of Western Electric by researchers interested in the relationship between productivity and work environment. Surprisingly, contact with the research team, rather than manipulations of the experimental variables resulted in improved productivity [23]. The Hawthorne effect did historically refer to behavioral changes (increased productivity) that derive from being the object of observation, rather than from the specific nature of the modifications made to the environment in which the study subjects operate, such as lighting in the historical example. Schwartz et al. recently described the “pure” effect of participation in another consumer setting [24]. In a study of residential consumers' electricity use, five post-cards were sent to a random sample of customers, notifying them at first and then reminding them of their participation in a study of household electricity use. Being reminded that they were in a study was associated with a 2.7% reduction in monthly use of electricity. When the intervention ended, energy consumption returned to pre-study values. Typically, the Hawthorne effect is quite transient and it disappears once study participation and observation of the subjects are stopped. Since its original description, the Hawthorne effect has been also reported in the field of sociology, psychology, as well as in several fields of clinical medicine. For instance, patients who were aware of being in a study had less post-operative knee pain than subjects who were unaware [25]. Being aware of ongoing monitoring was associated with improvement in decontamination hospital practices [26].

Nevertheless, we need to note that some features of our findings are not typical for the classic Hawthorne effect. Until the Randomization Visit, our subjects were not yet “participating” in the study, they were “candidates” for participation. Hence they were not “naïve” about the goal of the study (sleep extension) and its general finalities by receiving a fair amount of knowledge just from the screening informed consent alone. Further, there may have been a potential bias in the study admission procedure after screening as enrollment was contingent upon compliance with study requirements in terms of collection of information and documentation, in other words, of “good” behavior. It is possible that candidates, especially if committed to be accepted into the study, decided, either consciously or unconsciously, that in order to secure participation, they would strictly adhere to the requirements of recording their sleep, weight and blood pressure, and wearing wrist actigraphy for weeks; thus, they may have changed their behavior in anticipation of study entry. This participant selection procedure was necessary because poor adherence to study requirements *per se*, whether of behavioral nature or of the necessity to take a study drug (either active drug or placebo) is considered a negative predictor of study outcome. In the Canadian Amiodarone Myocardial Infarction Arrhythmia Trial, poor adherence to study medication, both active *and* placebo, was associated with a two-fold increase in relative risk of sudden death of 2.1 [27]. Finally, at variance with the classical Hawthorne effect that typically disappears once study participation and observation of the subjects are stopped, our subjects continued to participate and to be observed beyond randomization, yet the initial improvements in sleep, anthropometric and biochemical parameters were largely or completely gone without intervention about 4 months after screening.

Another issue is that, despite supporting evidence from a variety of sources, the existence of the Hawthorne effect is not universally accepted. Criticisms include that the original reports of this effect are “nebulous” and that the effect is both ill-defined and seldom reproducible [28]. To address these criticisms, clinical researchers are more often characterizing observations that would have been previously categorized as “Hawthorne” effect in a different construct called “research participation effect” to include the effects that may derive from patient preferences in the context of the various demands of clinical trials [29]. This may be especially true for the earlier stages of a trial prior to randomization, especially if more than a few days separate these stages and the study is of behavioral nature as in our case.

To further complicate matters, it is a widely accepted practice for ethical and regulatory purposes in randomized clinical trials to recommend to participants, regardless of group allocation, adherence to the “standard of care”. Thus, in weight loss trials, diet and exercise are prescribed in addition to drug and placebo, because these non-pharmacological interventions represent the currently accepted standard of care for obesity. The typical design of a randomized controlled trial does not allow dissecting out how much of the therapeutic effect may be due to the study drug *vs.* the implementation of the standard of care. This is one of the reasons why the therapeutic effect obtained in randomized controlled trials is seldom comparable to therapeutic effect observed in clinical practice, in which the standard of care is less consistently enforced.

Accepting the validity of the Hawthorne effect, detecting and characterizing its features in different experimental situations is important, especially for behavioral interventions. In clinical research, the Hawthorne effect might at times introduce confounding factors, whereas in clinical practice it may result in beneficial behavioral changes, otherwise difficult to obtain. This effect is operational in longitudinal studies, especially with behavioral interventions, and should be distinguished from the natural history, placebo, and treatment effects [30]. In our study, no “classical” placebo effect was present, as no study drug was administered per protocol. Furthermore, the general direction of the changes was congruent among the different parameters, consistently indicating clinical improvements. Regression to the mean, that is the well-known phenomenon according to which if a variable is extreme on its first measurement, it will tend to be closer to the average on its second measurement and *vice versa* cannot be completely excluded but it is unlikely, given that our measurement values generally were not extreme.

In addition to “research participation effect” or the Hawthorne effect, our experience might also be conceptualized based on another principle. In quantum physics, the “Observer Effect”, also known as the “Uncertainty Principle”, named after the German scientist Werner Heisenberg who first described it in the 1920's, states that measurements of certain physical systems cannot be made without affecting the very same systems they are meant to describe. In that sense the phenomenon that we have described and characterized is akin to the Heisenberg principle, and carries implications that may revolutionize clinical research as a discipline. We therefore caution that the “gold standard” study design which includes a ‘run-in’ period, previously regarded as neutral, may in fact markedly alter baseline measurements and therefore have important effects on the predetermined sample size and study outcomes, especially for behavioral studies. In spite of the common presence of an initial run-in period, especially in drug trials, there is little empirical evidence establishing its relevance [31].

Independent of the specific nature of the mechanism(s) at play, clinically meaningful improvements were achieved by simply being screened for enrollment into our Sleep Extension Study in sleep and glucose parameters in a chronically short-sleeping obese adult population. Remarkably, almost half of the subjects that had metabolic syndrome at screening were temporarily “cured” by the time they were randomized about three months later. A legitimate question therefore becomes how to reproduce these effects in clinical practice and, possibly, to maintain them over time. Even in pharmacological trials, where an active drug is administered to the intervention group, the importance of the ritual process and of the psychosocial context is being increasingly recognized [32]. The desire of harnessing this effect should, however, be balanced with the accepted right of properly consenting subjects; the inherent tension between these two factors poses challenges that deserve wide discussions by the scientific community. In prospective studies, whether of observational (i.e. cohort studies) or of interventional nature, the designation of the study “baseline” has important implications, as all subsequent modifications are compared to it. Conventionally, the baseline is considered the time of enrollment in case of a prospective cohort study, or the time of randomization. Little attention is usually paid to the characterization of study candidates at the time of the first encounter, other than for reasons of determining eligibility. Even less attention is subsequently devoted to the modifications that take place in the characteristics of the study population between the first encounter and the “official” baseline; in spite of having collected the data, seldom changes between screening and the official start in the study for those candidates that are eventually enrolled are analyzed and shown. This widespread and accepted practice introduces a number of biases that are currently not fully appreciated by investigators, peer reviewers, editors of academic journals, and regulatory agencies.

An inherent limitation of our study was that the nature of the study design did not allow for dissecting out of how much of the observed changes were due to an intentional effect, as compared to something non-intentional that we characterized in the context of the Hawthorne effect. Most likely, such a desirable study design does not exist for the reasons that we have enumerated earlier (i.e., akin to the Heisenberg Principle in Physics). No matter what term we elect to describe the “non-specific” changes observed in our study and in other studies, these perturbations to the investigational systems do occur and ignoring them, as it often has been the case so far, as compared to acknowledging and trying to understand them obviously is unacceptable. Additional limitation include that the sample size was relatively small and that the Intervention Group was slightly more obese than the Comparison Group.

In summary, discovering between screening and randomization “unintended” changes primarily in sleep and glucose homeostasis without specific intervention in an adult obese population has potential implications that may trespass the design of behavioral studies in the field of sleep to include any type of life-style behavioral modification Moreover, our findings have far-reaching implications in clinical research for the design and interpretation of randomized controlled trials in general, and, possibly, in clinical practice for the non-pharmacological management of glucose abnormalities in obese subjects.

## Supporting Information

Checklist S1 **CONSORT checklist.**
(DOC)

Protocol S1 (DOCX)
