# Supplementary material for: Hawthorne Effect with Transient Behavioral and Biochemical Changes in a Randomized Controlled Sleep Extension Trial of Chronically Short-Sleeping Obese Adults: Implications for the Design and Interpretation of Clinical Studies
Source: PLoS One. 2014 Aug 20;9(8):e104176. doi: 10.1371/journal.pone.0104176 (PMC4139265; doi:10.1371/journal.pone.0104176)
Supplement: Protocol S1 — (DOCX) [file pone.0104176.s002.docx]

Date: June 26, 2012

To:

Recommended by:

# Protocol Title: Chronic sleep deprivation as a risk factor for metabolic syndrome and obesity

Abbreviated Title: Sleep Administration Study

Identifying Words: Obesity, sleep deprivation, metabolic syndrome

Principal Investigator: Giovanni Cizza, M.D. Ph.D. MH.Sc., Staff Clinician, NIDDK

Medical Advisory Investigator: Giovanni Cizza

Associate Investigators: Amber Courville, R.D., PhD

Elizabeth de Jonge-Levitan, M.Sc, Ph.D, NIDDK (contractor)Wallace Duncan, Ph.D., NIMH

Gregor Hasler, M.D. Ph.D, University Hospital, University of Zurich

Megan Mattingly, RN, MPH, NIDDK, Study Coordinator

Nancy Sebring, R.D., NIH CC, special volunteer

Monica Skarulis, M.D., NIDDK

Collaborators: David Allison, PhD, University of Alabama at Birmingham

Nikhil Dhurandhar, PhD, Pennington Biomedical Research Center, Baton Rouge, LA

Richard Atkinson, PhD, Virginia Commonwealth University, Richmond, VA

Estimated Total Duration of Study: 7 years

Number of Subjects: 150 enrolled [approximately 300 screened]

300 external comparison

Kind of subjects: Obese men and women ages 18-50

Research Type: Randomized, comparison-controlled, clinical trial

Off-Site Protocol: No

Multi-Institutional Protocol: No

Investigational New Drugs/Devices: No

Ionizing Radiation Usage: Yes

Project Involves Durable Power of Attorney: No

**PRÈCIS**

OBJECTIVE: Obesity and chronic sleep deprivation have both become increasingly pervasive medical problems in recent years. The prevalence of adult obesity has doubled over the past 30 years and continues to increase. In addition, industrial societies attach an economic value to maximizing the waking period to the longest tolerable limit by sleeping as little as possible. Average sleep time has decreased over the last century by 2 hours. Chronically sleeping less has been associated with increased weight, endocrine and metabolic health risks including glucose intolerance, cardiovascular disease, and mortality. The possibility that the current epidemic of obesity and metabolic health risks may be partially related to insufficient sleep is now being recognized. The objective of this proof-of-concept controlled trial is to investigate the impact of increasing sleep time in chronically sleep-deprived, obese subjects.

STUDY POPULATION: 18-50 year old, obese (BMI 29-55) men and premenopausal women, chronically sleep deprived, recruited from the Baltimore-Washington metropolitan area. Chronic sleep deprivation will be verified by the use of sleep logs and the use of actigraphy before entry into the study. Secondary causes of sleep deprivation such as insomnia, psychological (depression), and medical conditions associated with poor sleep quality (including obstructive sleep apnea) will be exclusionary criteria.

DESIGN: This is a randomized, 12-month duration, comparison-controlled clinical trial of an extension of sleep up to approximately 7 hours and 30 minutes (Intervention Group) or continuation of habitual short sleep schedule (Comparison Group). The proposed treatment is an educational and behavioral intervention aimed at increasing sleep in a non-pharmacological fashion. The main analysis of the study will be to determine if additional sleep will result in a significant difference in body weight at the end of 12 months between the Intervention Group and the Comparison Group. In addition, we would like to establish whether 12 months of additional sleep will result in: a) a decreased prevalence of metabolic syndrome; and b) changes in the endocrine profile (i.e. inducing changes in leptin [increase] and ghrelin [decrease] opposite to the changes associated with chronic sleep deprivation). At the end of the 12-month intervention study (Phase 1, Efficacy [Randomized Phase] Study), all participants will be given information about the potential benefit of more sleep and encouraged to increase sleep time. Health teaching about proper nutrition and adequate exercise will also be provided at that time to the Intervention and Comparison Groups. All participants will be evaluated 6 months later to assess the effects of this intervention in a real-life situation, and offered participation in a three-year extension with semi-annual visits (Phase 2, Effectiveness [3 Year Follow-Up Phase] Study), for which matched external comparison subjects will also be recruited *ad hoc*.

OUTCOME PARAMETERS: body weight, average number of hours of sleep/night, fasting glucose and insulin, oral glucose tolerance test, leptin, ghrelin, adiponectin, other relevant endocrine and anthropometric measures, body composition, various metabolic parameters, food intake, energy expenditure, and quality of life measures.

# TABLE OF CONTENTS/OUTLINE

######

**INTRODUCTION 7**

*Increasing prevalence of obesity and metabolic syndrome*

*Increasing prevalence of chronic sleep deprivation*

### Chronically sleep-deprived subjects vs. naturally short sleepers

*Sleep apnea*

*Endocrine and metabolic consequences of sleep deprivation: laboratory studies*

*Endocrine and metabolic consequences of sleep deprivation: epidemiological studies*

*Sleep deprivation and personality traits*

*Sleep deprivation and the immune system*

*Obesity and the immune system*

*Obesity, sleep deprivation, and gene expression*

**OBJECTIVES 12**

*Research Question*

*Importance of the research question*

*Cost-effectiveness considerations*

Primary Objectives

Secondary Objectives

Tertiary (Exploratory) Objectives and Hypotheses

**STUDY DESIGN AND METHODS 15**

Study design

*Efficacy (Randomized Phase) Study*

*Effectiveness (3 Year Follow-Up Phase) Study*

*Sleep deprivation and the immune system: experimental approach*

ENDOCRINE AND METABOLIC ASSESSMENTS

SLEEP PARAMETERS

LIFESTYLE AND QUALITY OF LIFE

Study limitations and challenges

*Compliance with the study regimen*

*Blinding*

*Seasonal changes*

Potential confounders

### Contamination

*Pilot study*

*Control group*

*Length of efficacy study*

*Future studies*

**INCLUSION AND EXCLUSION CRITERIA 25**

## Inclusion criteria

*Exclusion criteria*

*Rationale for selection of study subjects*

**STUDY IMPLEMENTATION 28**

Flow Diagram

*Standardization and calibration of research procedures*

*Visits*

*Subject withdrawal*

*Phone contact*

*Schematic of study visits*

**ANALYSIS OF THE STUDY 37**

Stratification

*Metabolic syndrome*

*Age*

Sample size determination

*Changes in BMI*

Table 1

*Changes in ghrelin and leptin*

Table 2

Table 3

DATA SAFETY MONITORING PLAN 41

ADVERSE EVENT REPORTING PLAN 41

**HUMAN SUBJECTS PROTECTIONS**  42

*Recruitment*

*Compensation*

*Benefits*

*Risks and discomforts*

**RESEARCH USE AND DISPOSITION OF HUMAN SAMPLES AND DATA** 44

#### STUDY PUBLICATIONS 45

**ACKNOWLEDGEMENTS** 47

**REFERENCES**  48

##### APPENDIX 1 53

##### Time Line

##### Table of visits and procedures

##### APPENDIX 2 59

##### Description of procedures

Glossary of terms

**APPENDIX 3** 63

Qualifications of Investigators

**APPENDIX 4**  67

Compensation of subjects

INTRODUCTION

*Increasing prevalence of obesity and metabolic syndrome:* Obesity has become a major health problem because of its increasing prevalence and relation to multiple medical consequences [Bray 2004]. Adult obesity in America has more than doubled from 15% in the late 1970’s to 31% in 2000 [CDC 2004]. Even more alarming, over the same period, the prevalence of adolescent obesity has tripled from 5% to 15% [CDC 2004]. More than 280,000 deaths are attributable to obesity each year, and the cost to treat weight-related morbidity is estimated to account for 6.8% of healthcare costs in the United States [Wolf 1996]. Physical inactivity and easy access to high-fat foods are increasingly being recognized as major causes for obesity. Given the limited effectiveness of changes in lifestyle, marginally effective and not well tolerated medications for weight loss, and the fact that bariatric surgery is invasive, the prevalence of obesity and associated morbidity and mortality is on the rise. Obesity and insulin resistance are the hallmarks of metabolic syndrome, as recently defined by the National Cholesterol Education Program Adult Treatment Panel III:

- Increased waist circumference (102 cm in men and 88 cm in women)
- Elevated triglycerides (150 mg/dl)
- Reduced HDL cholesterol (<40 mg/dl in men and < 50 mg/dl in women)
- Elevated blood pressure (130/85 mm Hg or on treatment for hypertension)
- Elevated fasting glucose (100 mg/dl)

*Increasing prevalence of chronic sleep deprivation:* At the same time, chronic sleep deprivation is becoming another pervasive problem in today’s society [Bonnet 1995]. Industrial societies attach an economic and moral value to sleeping as little as possible to maximize the waking period to the longest tolerable limit. A historical trend in number of hours of sleep has been reported, with a progressive decrease over the last century from approximately 9 hours to 7 hours today, with 6 hours being common [Bonnet 1995]. Although habitual sleep duration varies greatly among individuals, the average sleep duration in the adult population is approximately 7.5 hours with a standard deviation of one hour [Aeschbach 2003]. It is estimated that, on average, adults need 7 to 8 hours of sleep per night, although there is no good test to establish the exact sleep needs of a given individual.

Interestingly, the epidemiology of sleep deprivation is reminiscent to that of obesity. Both conditions are more prevalent in lower socioeconomic classes and minorities [Van Cauter 1999]. It is estimated that up to one third of adolescents and young adults are chronically sleep- deprived. The consequences of chronic sleep deprivation are not trivial. A reduction in nocturnal sleep time of 1.5 hrs results in a reduction in daytime alertness of 32%, as measured by the Multiple Sleep Latency Test [Bonnet 95]. According to the National Transportation Safety Board, fatigue plays an important role in 57% of fatal accidents involving truck drivers [Bonnet 95]. Performances at 8 am after a night of sleep deprivation decline to a level comparable to performances with a blood alcohol level of 0.10% [Dawson 1997]. As an example, sleep deprivation has recently been recognized as a cause of medical errors in first year medical residents [Lockley 2004]. The possibility that the current epidemic of obesity and diabetes may be partly related to insufficient sleep is now being recognized.

*Chronically sleep-deprived subjects vs. naturally short sleepers:*  There is a wide range of individual differences in average sleep lengths in the general population. Short sleepers may be subjects that naturally need less sleep than others to be rested (“natural short sleepers”), although by several measures, for example the Multiple Sleep Latency test (MSLT), even these subjects are typically sleepier than other people, suggesting some degree of voluntary sleep deprivation. Of special interest are the findings reviewed by Dinges in 2004 suggesting strong inter-individual differences in the ability to perform and report sleepiness subjectively when subjected to chronic sleep restriction. Preclinical studies conducted in Drosophila demonstrate that a mutant line, called Minisleep, sleeps for one-third of the wild-type amount, performs normally in a number of tasks and carries a point mutation in a conserved domain of a specific gene, called the Shaker gene [Cirelli 2005]. The prevalence of “naturally short sleepers” in the human general population is not known, although it is reasonable to assume that these subjects are much less frequent than subjects who are chronically sleep-deprived. There is evidence that short sleepers usually do not experience daytime sleepiness or the need for extensive catch-up sleep on weekends [Monk, 2001]. Personality tests indicated that these subjects tended to be more extroverted, energetic, ambitious and less anxious but also aggressive at times [Hartmann, 1972] [Hasler, 2004]. The circadian pacemaker, which programs sleep duration, programs a shorter biological night in naturally short sleepers than in long sleepers [Aeschbach 2003].

*Sleep apnea:* It is estimated that there is a wide spectrum of sleep disorder breathing among adults. As an example, undiagnosed sleep disorder breathing, as indicated by 5 of more episodes of apnea or hypopnea per hour of sleep, has a prevalence of 9% in middle-aged women and 24% in middle-aged men. A recent overall estimate is that 5% of all adults suffer from sleep apnea [AHA Scientific Statement, in press]. The major predisposing factors for sleep apnea include male sex, obesity (especially upper-body obesity), craniofacial features predisposing to a narrow upper airway anatomy, and central factors mediating muscle tone control and breathing during sleep. Upper airway resistance syndrome (UARS) presents with symptoms that overlap those of obstructive sleep apnea but with differences, such as chronic insomnia, nocturnal awakenings, fatigue rather than sleepiness, confusional parasomnias, myalgia, depression and anxiety [Bao, 2004].

*Endocrine and metabolic consequences of sleep deprivation: laboratory studies:* Chronically sleeping less has been associated with health risks, including glucose intolerance, cardiovascular disease, and increased mortality [Ayas 2003]. In seminal studies conducted by Van Cauter et al., partial sleep deprivation (sleeping 4 hrs/night for 6 nights) in young, healthy men induced insulin resistance, as indicated by a 30% increase in glucose and insulin secretion [Spiegel 1998]. Additional endocrine changes observed as a result of sleep deprivation included increased evening plasma cortisol and decreased nocturnal TSH peak. Increased cortisol levels and increased sympathetic activity observed as a result of acute sleep deprivation are reminiscent of those of normal ageing, and may have long-term pathophysiological consequences [Van Cauter 2000]. It should be noted that some of these endocrine alterations are observed even in mild sleep deprivation. Even one night of partial sleep deprivation is associated with a 37% increase in mean cortisol the following day. Similar hormonal changes to two recent studies [Hasler 2004 and Taheri 2004] were shown in a randomized, 2-period, crossover clinical study conducted by Spiegel and colleagues [Spiegel 2004]. Twelve healthy men were studied after 2 days of sleep restriction or sleep extension under conditions of controlled caloric intake and physical activity. The period of sleep curtailment was associated with reduced levels of leptin (decrease, 18%; P=0.04) and increased levels of ghrelin (increase, 28%; P<0.04), both of which would stimulate hunger and possibly, weight gain.

*Endocrine and metabolic consequences of sleep deprivation: epidemiological studies:*  Recently, several epidemiological studies have reported that sleep loss is associated with increased body weight [Hasler 2004 and Taheri 2004]. In a population-based study of 1,030 subjects conducted at Stanford University, sleep durations below 7.9 hours were associated with increased body mass index (BMI). In that study, for a subject who slept an average of 5 hours, there was an estimated BMI increase over time of 4% compared to a subject who slept 8 hrs per night (BMI 31.4 vs. 30 respectively). In addition, changes in leptin and ghrelin opposite to what was expected as a consequence of increased BMI were observed: reduced sleep was associated with a 21% decrease in leptin and a 15% increase in ghrelin. Such changes are likely to stimulate appetite and food intake and to decrease energy expenditure, thus predisposing to obesity over time.

A community-based, 13-year prospective cohort study [Hasler et al. 2004] reported a negative association between sleep and obesity in young adults. Curtailed sleep duration was a strong and significant predictor of obesity (P<0.01). Every hour of more sleep cut the risk of obesity in half. Sleeping less than 6 hours at age 27 was associated with much greater chances of developing obesity later on (odds ratio: 8.2; C.I. 1.9-36.3]. Furthermore, change rate in sleep duration in minutes per year tended to be negatively associated with change rate in BMI per year after adjusting for sex (P<0.08).

Recently, cross-sectional data from the National Health and Nutrition Examination Survey Study (NHANES) reported that subjects between the ages of 32 and 49 years with sleep durations at baseline less than 7 hours had higher average body mass indexes and were more likely to be obese than subjects with sleep durations of 7 hours. [Gangwisch 2005].

A large prospective study of 1001 subjects from 4 primary care practices was recently published [Vorona 2005] reporting that a difference in sleep of 16 minutes/night, approximately 2 hours/week, translated into a difference in BMI of 10 units (from 22 to 32). Therefore, even a seemingly mild sleep loss can result over time in a considerable increase in weight. If this is the case, even a small increase in sleep time could be beneficial in terms of weight loss.

Sleeping longer hours, on the other side, may also be associated with some negative effects, including mortality. A recent review of the association between long sleep and mortality reported that sleeping more than 8 hours is associated with increased mortality [Youngstedt SD, 2004]. It should be noted that this epidemiological evidence should be confirmed in controlled study designed ad hoc. In 2004, Kripke et al. compared the rates of sleep problems in both long (more than 8 hours) and short sleepers (6 or less). This study addressed the question whether long sleepers reported more sleep complaints than midrange or short sleepers. Interestingly, a U-shaped relationship of sleep complaints with weekday total sleep time was reported. Subjects who slept 8 hours reported less frequent symptoms than long sleepers or 7-hour sleepers. It is unclear whether sleeping longer is a non-specific index of health problems as compared to a cause for increased morbidity. Whether older subjects may be more sensitive to the deleterious effects of sleeping more than 9 hours remains to be determined. The most important endocrine and metabolic changes observed in subjects that sleep 6 hours or less are summarized in the table.

|  | Mignot et al. | Van Cauter et al. | Hasler et al. |
| --- | --- | --- | --- |
| BMI | ↑ from 30 to 31.2 for  average 5 hrs of sleep  (N=1040) | NA | ↑ of 0.38+0.35/year (mean+SD) for an average of 5 hrs of sleep  (N= 591) |
| Leptin | ↓ 21.3% | ↓18% | NA |
| Ghrelin | ↑ 14.9% | ↑ 28% | NA |
| Glucose | NA | ↓ clearance 40% | NA |
| Plasma TSH | NA | ↓ 24h 44% | NA |
| Cortisol | NA | ↑ 23% of salivary cortisol | NA |

*Sleep duration and personality traits:* Previous research showed that aggressive personality traits including conduct disorder in children [Pine 1997], antisocial behaviors in adolescence [Hasler 2004], and hostility in adults are associated with increased BMI and persistence of weight problems [Mustillo 2003]. There is also preliminary evidence suggesting that adults who are chronically sleep-deprived score higher on the personality dimension “aggression” than those who do not deprive themselves of sleep. Aggression/antisocial personality may be a shared risk factor for both insufficient sleep and obesity [Hasler 2004].

*Sleep deprivation and the immune system:* There are bi-directional connections between sleep and the immune system [Irwin 2002]. Sleep is hypothesized to have a restorative effect on the immune function, whereas sleep deprivation has detrimental effects on immune cell number, function and cytokine production. Several cytokines show circadian rhythm. The pro-inflammatory cytokines, IL-1 and TNF-α are central to sleep regulation and have somnogenic effects [Benca 1997]. When administered, either peripherally or centrally, these cytokines induce non-REM sleep. After sleep deprivation, the expression of IL-1 and TNF-α increases in the brain, whereas inactivation of these two cytokines by specific antibodies inhibits spontaneous sleep. Similarly, anti-inflammatory cytokines such as IL-4, IL-10, and IL-13 or TGFß-1, which decrease the levels of IL-1 and TNF-α, inhibit non-REM sleep. Although IL-1 and TNF-α play an important role in sleep regulation, many other cytokines and hormones, including EGF, fibroblast GF, interferon α and other substances may induce sleep. Changes in IL-1 and TNF-α induce a series of downstream events, including activation of nuclear factor k-B, a gene transcription factor. This increase in turn up-regulates the expression of COX-2, adenosine A1 receptor and NO synthase and release of GHRH.

Cytokines alter sleep depth and intensity. IL-10 and TNF increase delta sleep and IL-10 inhibits slow-wave sleep in animals. Partial sleep deprivation, either during the late or early part of the night induces reversible changes, including a significant reduction of cellular immunity, as indicated by NK activity, and changes in natural immunity. Levels of IL-6, another pro-inflammatory cytokine, change across sleep stages.

*Obesity and the immune system:* The adipocyte is a hormone- and cytokine-secreting cell [Wisse 2004]. In addition, macrophages and other immune cells tend to migrate, following an unknown stimulus, to the adipose tissue and to aggregate into giant cells. This is clinically relevant, as chronic sub-clinical inflammation is one of the mechanisms leading to increased cardiovascular morbidity and mortality. Additional links between the immune and the adipose tissue include the fact that white adipose tissue and bone marrow both derive from the mesoderm.

Several cytokines have metabolic effects. Thirty percent of circulating IL-6 is derived from the adipose tissue; IL-6 is the single most important factor controlling hepatic acute-phase response and the liver production of CRP, one of the most important markers of metabolic syndrome. TNF-α, which is produced by macrophages within the adipose tissue, causes insulin resistance at the level of the adipocyte via inactivation of both the insulin receptor and the insulin receptor substrate 1, with consequent diminished activation of phosphoinositol-3-kinase, a second messenger that governs most of the insulin’s metabolic effects. In addition, TNF-α elevates free fatty acids and reduces adiponectin secretion from adipocytes. As obesity and chronic sleep deprivation are both associated with changes in cytokine levels and the activity of the immune system, a characterization of this system is warranted in the current study.

**OBJECTIVES**

*Research Question*

If sleep deprivation is associated with increased weight, what would be the impact of increasing sleep time in obese, chronically sleep-deprived subjects?

*Importance of the research question:* To address this question, we propose to conduct a proof-of-concept, prospective, interventional, randomized, 12 months-duration study of sleep administration in subjects who habitually sleep less than approximately 6 1/2 hours per night. Eighteen to 50 year old chronically sleep-deprived healthy obese men and premenopausal women will be randomized to sleeping up to approximately 7 hours and 30 minutes(Intervention Group) or to continuing with their habitual short sleep schedule (Comparison Group) (Phase 1: Efficacy [Randomized Phase] Study). At the end of the 12-month period all subjects in both groups will be encouraged to increase sleep up to a maximum of approximately 7.5 hours a night as part of a health education session on sleep, nutrition, and exercise. During the Effectiveness (3 Year Follow-up) Study Phase 2, the residual effects of such recommendations will be further evaluated in a “real life” situation at 6 months intervals for a total of 36 months

We think that such a study would be relevant for the following reasons: a) although there is evidence, which we have summarized, of the health consequences of chronic sleep deprivation, the effects of correcting sleep debt in a relatively large sample in the context of a controlled trial have not been studied; b) from a public health perspective such an intervention would be safe, inexpensive, and, given the epidemiology of chronic sleep deprivation, directed to less affluent strata of the population and to minorities; c) if positive, such a seminal study could foster future research towards the relationships between sleep patterns and body weight regulation. The research question proposed is novel, as to the best of our knowledge no interventional prospective studies of this kind have ever been conducted.

*Cost-effectiveness considerations:* The 12 month intervention study (Phase 1) will be aimed at showing the *efficacy* of implementing additional night time sleep, and the impact of a sleep intervention on the prevalence of metabolic syndrome and on circulating levels of leptin and ghrelin, in optimal, controlled circumstances. The 36-month post-study period (Phase 2), will assess the *effectiveness* of this health care intervention when used under usual, every-day circumstances. The efficiency or cost effectiveness of this intervention is likely to be very high, given the minimal amount of resources necessary to implement such intervention. Finally, the intervention proposed, sleep administration, is readily “available”.

Primary Objectives:

To determine the extent to which it is possible to implement sleep “prescriptions” and improve habitual sleep durations over time in chronically sleep-deprived subjects.

To establish whether increased habitual sleep durations for 12 months in obese, sleep-deprived, 18 to 50 year old men and women in a randomized, controlled fashion result in the following:

1. Significantly lower BMI at 12 months vs the comparison group
2. Decreased prevalence at 12 months of the metabolic syndrome

Secondary Objectives:

To establish whether 12 months of sleep administration in a randomized, controlled fashion to obese, sleep-deprived, 18 to 50 year old men and women will result in:

1. After 12 months of treatment fasting **ghrelin** will be fifteen percent or more **lower** in subjects randomized to increased sleep, compared to subjects randomized to continuing habitual short sleep.

Null hypothesis: mean 8 am fasting ghrelin levels after 6 months of sleep administration will be similar (within 15% or less) in the intervention group, compared to the comparison group.

1. After 12 months of sleep treatment fasting **leptin** will be 20 percent or more **higher** in subjects randomized to increased sleep, compared to subjects randomized to continuing habitual short sleep.

Null hypothesis: mean 8 am fasting leptin levels after 12 months of sleep administration will be similar (within 20% or less) in the intervention group, compared to the comparison group.

Tertiary (Exploratory) Objectives and Hypotheses:

Objective 1: To observe:

a) Whether a 12-month trial of intentional sleep increase results in a stable change in sleep pattern that persists over the following six months

b) Whether such a sustained increase in sleep duration is reflected by decreases in BMI

c) The impact of a brief session of behavioral intervention (sleep hygiene, diet, and

exercise) administered at 6 month intervals for a total of 3 years on body weight, endocrine, immune, and metabolic parameters in an outpatient ambulatory sample of a cohort of previously obese, sleep-deprived subjects who participated in a controlled, 12 month study of sleep extension.

Objective 2: To observe:

1. The impact on sleep habits of patient education about sleep delivered in a way that would be feasible in a community clinical setting
2. The extent to which participation in a 12 month sleep trial prior to sleep education would impact the effectiveness of sleep education (i.e. the intervention group can be differentiated from the comparison group during the Effectiveness [3 Year Follow-up] Phase)

Objective 3: To observe the impact of sleep administration on the immune system.

Hypotheses:

a) Twelve months of sleep administration will be associated in the intervention group with a decrease in pro-inflammatory cytokines and an increase in anti-inflammatory cytokines compared to baseline.

b) At the end of 12 months, levels of pro-inflammatory cytokines will be lower and anti-inflammatory cytokines higher in subjects randomized to the Intervention Group, compared to subjects randomized to the Comparison Group.

Objective 4: To observe the relationship of sleep deprivation and sleep administration to personality factors:

Hypotheses:

1. Aggression scores (NEO), hostility scores (Cook-Medley), and the prevalence rate of conduct disorder (CIDI) will be higher in the study sample of obese subjects who are sleep deprived relative to the general US population.
2. Non-response of body weight to sleep intervention in subjects high in aggression/hostility will be associated with lack of compliance with the study procedures.

# STUDY DESIGN AND METHODS

# *Sleep intervention study: Study Groups*

|  | Baseline | Part 1 Efficacy (Randomized Phase) Study | Part II  Effectiveness (3 Year Follow-up Phase) Study | |
| --- | --- | --- | --- | --- |
| Study groups | Month 0 | Month 12 | Study  groups | Month 18-48 |
| Intervention Group  90 min of additional sleep at night | N= 100* | N =72 or more | All subjects receive health education about exercise, nutrition, and increased sleep with encouragement to increase sleep up to maximum of 7.5 hours/night | N = 61 or more |
| Comparison Group  continue habitual short sleep schedule | N = 50 | N= 37 or more |  | N = 31 or more |
| External Comparison subjects | NA | NA | External Comparison subjects  N=300** |  |

* to allow for a 15% dropout rate between Baseline and Month 6, and Months 6 and 12

** individually matched to original subjects based on age, race, gender, BMI, menopausal status and sleep duration

# *Study Design*

*Efficacy (Randomized Phase) Study*

This is a randomized, comparison-controlled, clinical trial of additional sleep per night (Intervention Group) or continuation with habitual short sleep schedule (Comparison Group). When subjects have completed the screening process and study entry process and sleep habits have been characterized, they will be randomized to the Intervention or Comparison Group.

The Efficacy (Randomized) Study Phase will be a 12-month trial of sleep administration. At month 0 there will be an overnight visit for collection of initial data. Subjects will return to NIH Clinical Center at one, two, four, six, eight, and ten months for interim visits and at month 12 for collection of the end-point data (see appendix 1 and table).

Unbalanced randomization will be employed with 2:1 ratio of Intervention Group to Comparison Group to maintain sufficient sample size in both groups while collecting more information on the group of greater interest, the active treatment group.

*Intervention Group*: Subjects in the Intervention Group will be instructed to attempt to increase sleep up to the difference between their current sleep and approximately 7 hours and 30 minutes (e.g. if a subject habitually sleeps 5 hours a night, they would be instructed to increase their sleep by 2.5 hours in order to make up the deficit, and a subject who sleeps 6 hours, an additional 1.5 hours). The additional sleep may take place preferably in the evening, but if that is not feasible, it can also take place in the morning, as an extension of the primary night sleep period, based upon the constraints of each individual participant. Participants will be allowed a two-week period in which to progressively build up the sleep increase over their previous sleep time.

Those subjects randomized to the Intervention Group who are willing and able to increase their sleep time up to 7.5 hrs will be encouraged to do so, as sleep debt should be repaid in full for our intervention to be maximally effective. However, since this approach may not be feasible for all subjects, we require participants to agree to attempt to sleep 90 additional minutes (approximately 60 minutes for those subjects who sleep approximately 6 hours and 30 minutes). Because of existing evidence [Kripke, 2002] that longer sleep hours may have negative effects, subjects will not be encouraged to sleep longer than 7.5 hours.

Sleep Education and Coaching: We will utilize an educational and behavioral intervention. In a session tailored to their particular life circumstances and reasons for electing to sleep less than 6 1/2 hours, each subject will receive individual coaching on sleep hygiene and strategies to increase sleep. Based on the information gathered by the Morningness-Eveningness questionnaire at the screening visit, their circadian tendencies will be considered in recommending whether to attempt to go to bed earlier or to get up later in the morning. Subjects will be encouraged to maximize exposure to bright natural or artificial light early in the day, and minimize it later in the day, in order to assist their “body clock” to be ready for sleep at night.

Subjects for whom a hectic life style interferes with regular and sufficient sleep will be engaged in examining their daily schedule and strategizing changes in routine to allow for increased sleep as a priority. Everyone will be encouraged to establish a regular sleep schedule, with a set bedtime and awakening time. Napping will be discouraged, and if a subject does nap, it should be for no more than 45 minutes and not after 5 p.m. in order not to interfere with nighttime sleep.

Subjects will be instructed to limit stimulants in the evening before bedtime. We will recommend that they not drink excessive amounts of caffeine or alcohol within 6 hours of bedtime, and that they avoid heavy, spicy, or sugary food within 4-6 hours of bedtime. Strenuous exercise within 2 hours of bedtime will be discouraged, as well as nicotine in the 2 hours preceding bedtime.

The importance of the sleep environment will be discussed, with attention to comfortable bedding, temperature, and ventilation, absence of disturbing noise, and elimination of interference by light. Subjects will be advised to reserve the bed for sleep and sex.

Watching television in bed before sleep will be discouraged, with suggestion that the TV be in another room, or at least turned off at bedtime. Listening to the radio to fall asleep will be suggested as an alternative. Subjects will be assisted in devising a bedtime routine which may include such sleep helps as a light snack (warm milk or other foods high in tryptophan), relaxation techniques (yoga, deep breathing), pre-sleep rituals (a warm bath, reading), and not entertaining “worries” at bedtime. They will be encouraged to use the same strategies when awakening during the night. (Sleep hygiene hints adapted from University of Maryland Sleep Disorders Center web site: [http://www.umm.edu/sleep/sleep_hyg.html])

When feasible, we will encourage the involvement of the subject’s sleep partner or other household member to be aware of and reinforce the change that the subject is attempting.

Subjects may be called after the first week of the sleep increase, in order to address questions or problems that have come up and to reinforce coaching and encourage change. They will have a phone number to reach the study team as needed.

*Comparison Group:* Subjects randomized to the Comparison Group will be permitted to maintain their habitual sleep schedule, without alteration in their typical short hours of sleep. In order to pay the same level of attention to them as to the treatment group, they will be subjected to the same coaching in terms of following the protocol and carefully documenting their sleep and feeding habits. They will also receive calls and assistance with questions and concerns and encouragement regarding study participation and compliance with study requirements. Although we hypothesize that increased sleep would be beneficial in reducing BMI or preventing BMI increase over time, that has not been established; therefore, the principle of clinical equipoise is respected. In order to examine the effect of sleep administration, the presence of a comparison group who does not increase sleep is important. We will, in the effectiveness (3 Year Follow-up) phase of the study, offer the Comparison Group subjects the benefit of sleep hygiene teaching and encouragement to increase sleep.

*Effectiveness (3 Year Follow-up Phase) Study*

In the Effectiveness (3 Year Follow-up Phase) Study Phase we will assess the stability of the sleep duration changes, if any, obtained during the Efficacy (Randomized) Study Phase. We will also determine the effectiveness, in natural conditions in a real-life situation, of instruction to increase sleep along with other lifestyle changes, and if that instruction is more effective in subjects with prior sleep coaching. We will recall subjects at 18 to 48 months for follow-up assessments to evaluate if there is a lasting impact of sleep and other lifestyle recommendations in the subjects’ natural home environment. At the 12-month visit that ends the Efficacy (Randomized) Phase of the study, we will provide information to subjects in both groups about the potential benefits of longer sleep duration, along with nutrition and exercise education. Although the final results of the Efficacy (Randomized) Phase will not be available at the time of this visit, we will encourage all participants, in both the Intervention and Comparison Groups, to increase sleep duration up to a maximum of 7.5 hours. This design allows us to make the potential benefits of education regarding increased sleep available to all participants.

The Effectiveness (3 Year Follow-up) Study Phase will last 36 months. Thus, a subject’s participation could extend over a 48-month (4 year) period, comprising Efficacy (Randomized Phase) (12 months) and Effectiveness (36 months follow-up) Phases. Subjects will return at 6-month intervals during the Effectiveness (3 Year Follow-up) Phase. At alternate visits that are 1, 2, and 3 years after the end of the Efficacy (Randomized Phase) Study, a comparison subject (external comparison) will be recruited to match each subject for that visit and allow between-group comparison. The visits at 6, 18, and 30 months of the extension are “Interim Visits,” the visits at 12, 24, and 36 months of the extension are “Main Visits.” for which external comparisons will be recruited to match the subjects.

External comparisons will be individually matched to each original study participant based on age (3 years), BMI (2 units) gender, race, and menopausal status. In addition, external comparisons will be individually matched to each original study participant (case) based on sleep duration similar to the sleep duration of the matched case at the time of recruitment in the original study (within 30minutes). To minimize the so-called “Hawthorne Effect” of inducing by the mere means of observation changes in the health behaviors (diet, exercise, sleep) of the external comparisons, these subjects will be screened and serve as external comparisons preferably not more than once or twice.

Through the Effectiveness (3 Year Follow-up) Study Phase we will analyze the changes in metabolic and endocrine measures and BMI between Month 12 and Month 18-48. We will also perform an analysis to discern differences between the subjects who were previously randomized to the Intervention and Comparison Groups to ascertain the potential effect of the previous group allocation.

*Sleep deprivation and the immune system: experimental approach*

As cytokines have pleiotropic actions, it is important to contextually measure as many cytokines and immune products as possible involved in the immune response to sleep deprivation. We propose to measure 8 am plasma levels of several pro-inflammatory (including IL-1, TNF-α, and IL-6) and anti-inflammatory (IL-4, Il-10, IL-13) cytokines in study participants at the beginning and at the end of the intervention study. In collaboration with Dr. Phillips’ laboratory, cytokines will be measured by the use of recycling immunoaffinity chromatography, a technique that allows, by recycling of the same sample run over different columns each coated with a different antibody, the measurements of many cytokines and other analyses by using a minute amount of biological sample [Castle 2003]. For statistical purposes, in order to minimize the possibility for type 1 (false positive) and type 2 (false negative) results, we will use a composite end-point with a component for the pro-inflammatory and a component for the anti-inflammatory cytokines. As the field of cytokines is rapidly expanding, we are planning to add measurements of these new cytokines, if relevant to our research question, to our list.

Commercial assays have not been developed for all of the cytokines of interest. When they are available, such assays may not have the sensitivity required by our study. For these reasons all of our cytokine assays will be analyzed, blinded to treatment group, in the lab of Dr. Terry Phillips. The special qualifications of Dr. Phillips are listed in Appendix 3.

ENDOCRINE AND METABOLIC ASSESSMENT**S**

Several endocrine changes have been described as the result of chronic sleep deprivation, including a decrease in leptin and an increase in ghrelin that together would induce increased appetite and food intake. Additional changes related to sleep deprivation include a subtle increase in cortisol levels and a decrease in GH. It is possible that other sleep induced abnormalities exist and may not have been yet documented.

*Metabolic syndrome:* Metabolic syndrome is characterized by a constellation of metabolic risk factors associated with increased cardiovascular disease. The metabolic syndrome is defined by the presence of three or more of the following risk factors:

1. Waist circumference >102 cm (>40 in) in men and >88 cm (>35 in) in women
2. Triglycerides concentration ≥150 mg/dl,
3. Serum HDL cholesterol concentration <40mg/dl in men and <50 mg/dl in women,
4. Blood pressure ≥130/85 mmHg, and
5. Fasting glucose $\geq$100 mg/dl

In addition, although it is not part of the formal definition of metabolic syndrome, a pro-inflammatory state, as indicated by increased CRP or cytokines, seems to be part of this syndrome.

*Fasting leptin, ghrelin, and adiponectin:* Leptin, ghrelin, adiponectin, ACTH, TSH, FT4, GH and IGF-1 will be measured at 8 am in fasting conditions. Urinary free cortisol (UFC) will be measured in a 24-hour urine collection.

*Glucose metabolism*

Insulin resistance and diabetes are often associated with overweight as well as sleep deprivation. We will screen for the conditions as well as examine changes in the measures with sleep increase.

Fasting insulin and glucose with HOMA and Quicki computation

Oral glucose tolerance test with Hgb A1c

*Body composition*

Body composition measures provide additional information on fat and lean mass that measurements of height and weight cannot provide.

Anthropometric measurements (neck circumference, waist, hip, waist/hip ratio)

Plethysmography by air displacement method “[BOD POD]”

DEXA (fat mass, lean mass)

CT of the abdomen (L2-L3 and L4-L5 slices) for central fat

Bioelectric impedance analysis

*Energy expenditure*

Indirect calorimetry will be performed to assess the amount of energy expenditure and the effects of sleep increase or its lack (control group) on the parameter.

*Cardiovascular*

Resting heart rate and blood pressure

ECG

Fasting lipid panel (total cholesterol, triglycerides, HDL, LDL)

*Inflammatory markers*

High sensitive C reactive protein

Cytokine panel

SLEEP PARAMETERS

We will determine sleep patterns by sleep questionnaires, sleep diaries, activity watches, and an overnight polysomnogram (PSG).

It is likely that the number of sleep hours will be different between working days and weekends. To increase the feasibility of the study, we will allow subjects to sleep longer on weekends. We will measure not only habitual total sleep time, but also average weeknight (Sunday-Thursday) or work night and average weekend (Friday-Saturday) or non-work night sleep durations. However, in our analysis, there will be specific parameters that will capture the total number of hours and minutes that a subject sleeps in a given week.

LIFESTYLE AND QUALITY OF LIFE

Several dimensions of health-related quality of life (HRQL) will be assessed in an exploratory fashion. There is a large body of literature indicating that obesity is associated with poor HRQL. In contrast, there is little information on the effects of sleep administration on these parameters, and the information available is mainly anecdotal. A population-based study found no association between health-related quality of life, measured by the Quality of Well-Being (QWB) scale, and greater habitual sleep duration [Jean-Louis G, 2000]. We will assess chronic pain syndromes with attention to duration and intensity of pain and analyze the information in relation to sleep duration. Results of a study of adolescents with chronic pain [Palermo 2005] suggested a relationship between pain and sleep disturbances, and that the sleep disturbances are linked to depressive symptoms and reductions in daily functioning and quality of life.

## This controlled trial offers the valuable opportunity to collect important information in a prospective fashion. This is particularly important for measures such as HRQL, which, by definition, are subjective. It is possible that quality of life may change, as a result of sleep administration, earlier than the endocrine and metabolic parameters, providing an additional rationale for such intervention. To maintain the feasibility of the study without burdening participants and staff, only essential information related to the most important dimensions of HRQL will be explored. Such information may, however, guide the design of future studies devoted to specifically explore such issues.

We would also like to get an estimate of how much physical exercise the subjects engage in to control for it; as historical recollection of physical exercise is at best imprecise, we would like to use the level of physical conditioning, as assessed in the Cooper test, as an indirect index of their habitual level of physical exercise. We will use an activity monitor worn at the waist (Actical) to record activity for two-week periods between visits.

Quality of Life SF-36 (multidimensional health assessment)

SIP (Sickness Impact Profile) [Bergner 1981]

McGill Pain Questionnaire [Melzack 1976]

Quality Life Enjoyment and Satisfaction Questionnaire

[Endicott 1993]

Sexual Satisfaction Analog Scale

Appetite 10 cm visual analog scales (8 am fasting)

Caloric intake Food records with caloric calculation

Food preference Questionnaire; food records with macronutrient analysis

Caffeine intake Food/beverage records

Daytime sleepiness Stanford Sleepiness Scale

Epworth Sleepiness Scale

(PVT) Psychomotor Vigilance Task

Physical Activity Activity monitor (Actical)

Work and Home Activities Survey (Block)

MAQ (Modifiable Activity Questionnaire)

IPAQ (International Physical Activity Questionnaire) AAFQ (Arizona Activity Frequency Questionnaire)

Physical fitness (Cooper test, Step Test)

Pedometer readings

Neuropsychological testing Grooved Peg Board Test (visual-motor

coordination)

California Verbal Learning Test (CVLT)

(verbal memory abilities) [Halbach

2003]

Symbol Digit Modalities (mental efficiency)

[Rosano 2005]

Wechsler Abbreviated Scale for Intelligence

Boston Naming Test

Tower of London

Rey Complex Figure

Stroop Test

Wisconsin Card Sorting Test

FAS

Trail Making Test

Continuous Performance Test

Iowa Gambling Test

Mood and anxiety Hamilton Depression and Anxiety Rating

Scales

Beck Depression and Anxiety Inventories

Personality Traits Neo Five Factor Inventory

Cook Medley Hostility Inventory [Cook 1954]

CIDI: Conduct disorder; Childhood background

Psychiatric Diagnoses SCID (Structured Clinical Interview for

DSM-IV Diagnoses

## Study limitations and challenges

## Compliance with study regimen: The most difficult challenge is to ensure and monitor adequate compliance with the study regimen (i.e. increasing sleep duration in the intervention group). This will be achieved by enrolling motivated subjects who indicate willingness to increase habitual sleep times. We will pre-screen prospective study participants and enroll them for two weeks in order to assess their ability to complete diaries and questionnaires and adhere to the study guidelines. Following randomization, the Intervention Group will have a two-week period in which to increase their sleep by 90 minutes, preferably at night. In addition, to ensure optimal compliance, the study coordinator will promptly review the sleep diaries that study subjects will fill for the two weeks preceding each visit. The Study Coordinator will maintain weekly phone contact with subjects in order to assess compliance, provide encouragement and support of efforts, reinforce instructions, answer questions, and address concerns. Subgroup analysis will be done to control for different degrees of compliance with requirements of increased sleep at night. Similarly, for the Intervention Group, subgroup analysis will be used to analyze separately those subjects who slept more than they were supposed to.

## Compliance with filling out the sleep diaries will be measured first by an examination of the record for completeness and plausibility. The definitive measure of compliance for the diary will be the agreement with the activity watch data. Compliance with the study requirement of increasing sleep duration (Intervention Group) or maintaining habitual sleep pattern (Comparison Group) will be based on the average sleep duration over the course of the week. Because of the amount of increased sleep that previous studies indicate may be needed to effect the change in ghrelin and leptin that would be significant with the number of subjects in our study, we have established that subjects must show an average increase of 60 minutes/night to be included in the primary analysis. Subjects who increase their sleep by an average of 30 minutes or more per night will still continue to be active in the study and will be included in other analyses.

## When it is possible, we will, with the permission of the subject, involve the subject’s sleep partner or another member of the household as an observer and reporter. We recognize that for our measures of sleep, activity, and food intake, we are depending in part upon the ability of a subject to self-monitor and self-report. In order to enhance the accuracy of information, we will, with the consent of the subject, obtain information from another person and enlist that person to support and reinforce the subject’s endeavor to maintain the study regimen to which they are assigned.

##

*Blinding:* Blinding of treatment allocation is not possible in this study. This may pose a substantial problem, as once subjects learn that they have been randomized to a group different from their expectations and hopes they may withdraw consent. In addition, some subjective endpoints exist with this study that are inherently susceptible to the placebo effect. An effort will be made to enroll only subjects who are highly motivated and committed to participate in the study, independently of study allocation. In addition, biological samples will be run blindly to treatment allocation; and, to the extent possible, evaluations that imply a certain level of subjectivity (i.e. psychological and quality of life measures) will be conducted by research team members who are blinded to the subject’s group assignment.

*Seasonal changes:* Seasonal changes can affect many of the parameters measured in this study, including several endocrine and metabolic parameters, physical activity [Plasqui 2004], and sleep [Wehr 1998 and Kohsaka 1992]. Leptin levels, measured as a single fasting sample, do not seem to change in relation to seasons [Cizza 2005]. Because of the duration of the recruitment and the treatment period, subjects will be studied throughout the year. In addition, we will adjust for season during the data analysis.

## Potential confounders: We will encourage subjects to maintain their previous lifestyles during the Efficacy (Randomized) Phase, especially in regard to diet and exercise, and adhere to change or lack of change in their sleep pattern as indicated by the protocol. The randomized study design should provide for the confounders to be distributed similarly between the two groups.

### Contamination: To avoid treatment contamination between groups within the study, a phenomenon known as drop-ins (i.e. subjects who are randomized to the Comparison Group may decide to change their habits and sleep more because they have learned about the potential advantage of longer sleep), we will make sure that the two groups (Comparison and Intervention) will be seen in the clinic on different days.

### Pilot study: The first 10 subjects will be studied for the first month to assess the burden associated with study requirements (filling out questionnaires and diaries, wearing the activity watch, achieving an increase in sleep time). If the requirements are excessive and translate into poor compliance or subjects’ discomfort, the protocol will be modified to facilitate feasibility while maintaining scientific validity.

*Control group*: The choice of an appropriate control group is even more challenging in this study because of the specific nature of the intervention. In previous discussions, we considered asking a control group to spend additional quiet time in bed without sleeping, but we decided not to do so for several reasons. Subjects who chronically sleep deprive themselves would be more likely to sleep inadvertently, rather than spending time in bed awake, thus dropping into the Intervention Group. In addition, such a requirement would have made study compliance for this group arduous without providing any foreseeable clinical benefits. Finally, Hasler et al (2004) showed in their study that time in bed, and not sleep time, was associated with BMI changes. Therefore, we have settled for a Comparison Group, realizing this is an inherent limitation of the current study.

For the Effectiveness (3 Year Follow-up) Study Phase. during which the originally enrolled subjects from both Intervention and Comparison groups are encouraged to increase sleep and adopt healthy eating and exercise behaviors in a real-life situation, we have chosen to recruit, for control purposes, a new subject to match each of the original subjects at each visit. These new subjects will be matched to the original subject’s gender, race, age (3 years), BMI (2 units), and sleep at the time of enrollment (within a 30 minute window). These newly recruited external comparison subjects will thus be similar to the original subjects but will never have been exposed to the intervention regarding sleep, diet and exercise.

*Length of efficacy (randomized phase) study:* We chose 12 months, as compared to a shorter or longer duration, as the time period for the Efficacy (Randomized) Phase of the study, in order to allow time to show a robust effect on BMI while still minimizing the burden on the subjects who are being asked to make sizeable changes in their routine and devote considerable time to recording information, and also in order to not prolong the interval for the Comparison Group to be without benefit of a potentially beneficial intervention.

*Future studies:* Future studies should address the impact of increases in habitual sleep time in **sleep-deprived subjects with normal weight**. We realize that attempting to decrease body weight by administering sleep to obese subjects is an ambitious goal and as such may not be achieved. But even if subjects did not significantly lose weight as a result of sleep administration in this study, we feel that a follow-up study of sleep administration in sleep-deprived subjects of normal weight would still be warranted. In other words, in principle, sleep administration might be effective but the effect may not be sufficient to combat established obesity. Nonetheless the same intervention may be sufficient to prevent the development of obesity in normal weight subjects. If, however, the result is positive and administering sleep decreases body weight, a study with control subjects lying in bed but not sleeping would provide information on the sleep vs. rest effect on BMI and metabolism. Another future study of importance would be to administer sleep to **chronically sleep-deprived obese children**.

**INCLUSION AND EXCLUSION CRITERIA**

## **Inclusion criteria**

1. 18 to 50 year old obese men and premenopausal women
2. BMI between 29-55
3. Chronically (for more than 6 months) sleep-deprived, defined as sleeping on a regular basis less than or equal to approximately 6 ½ hours/night by history and objective devices (wrist activity monitors and sleep logs)

## **Exclusion criteria**

## Diagnosed sleep disorders including:

## Chronic insomnia

## Untreated sleep disordered breathing (sleep apnea at a level of severity [using standardized criteria for measurement], or diagnosed UARS [upper airway resistance syndrome] that would impair the ability to increase sleep duration [Intervention Group] or maintain sleep duration [Comparison Group]. CPAP treatment that has been in place for 3 months or more and improves sleep is acceptable)

- - - - 1. Restless leg syndrome or periodic limb movement disorder
        2. Parasomnias (including REM sleep behavior disorders, confusional arousals, sleep terrors, sleepwalking, sleep violence)
        3. Primary bruxism is allowed as long as it does not interfere with the ability to sleep an additional 90 minutes a night
        4. Narcolepsy
        5. Central apnea.
      1. Unstable weight (voluntary losses in BMI greater than 5% over the past 6 months); currently being enrolled in a weight loss program
      2. Untreated or uncontrolled diabetes
      3. Severe uncontrolled hypertension
      4. Other chronic organ disease diagnosis including:
         1. COPD
         2. Chronic cardiac arrhythmia requiring treatments
         3. Gastro-esophageal disorders associated with sleep-related symptoms.
      5. Medications
         1. chronic use of prescription or over-the-counter medications known to affect sleep (e.g., systemic steroids, NSAIDs)
         2. current anticonvulsant therapy
      6. Chronic fatigue syndrome and fibromyalgia

(8) Acromegaly, hypothyroidism (unless on a stable replacement dose of thyroid hormone), Cushing disease or other endocrine disorders known to affect sleep

(9) Poorly controlled major depression (subjects who have been on a stable pharmacological antidepressant treatment for 3 months and are in remission without substantial weight gain are eligible).

(10) Other current DSM-IV diagnoses, including:

- - - - 1. Eating disorders such as bulimia nervosa and binge eating disorder
        2. Anxiety disorders such as PTSD and panic attacks
        3. Mania
        4. Schizophrenia.

(11) Medication and substance abuse such as excessive alcohol consumption or drug abuse or dependence that may pose a threat to compliance

(12) Being a rotating worker, shift worker (working evenings or nights), or long distance commuter (more than approximately 90 minutes each way), traveling frequently outside of time zone; being in an occupation that may require special vigilance such as driving a truck, bus, or cab; operating heavy machinery; being a pilot or air traffic controller

(13) Being likely to move to a different geographical area during the study

(14) Having a sleep partner that would make compliance with study requirements difficult

(15) Pregnancy and lactation

(16) Menopause

(17) Chronic excessive caffeine use (habitual intake of more than 500 mg/day)

(18) Any condition that in the opinion of the principal investigator makes study participation and compliance problematic.

***External comparison subjects for Effectiveness (3 Year Follow-up Phase) Study***

Subjects recruited as external comparisons for the visits in the Effectiveness (3 Year Follow-up) Study Phase must meet the same criteria for inclusion.

1).*Rationale for selection of study subjects*

Study subjects will be young and middle-aged obese men and premenopausal women who are chronically sleep-deprived. This sample will be representative of the target population because an effort will be made to recruit subjects of lower SES and minorities, as this is the population in which chronic sleep deprivation seems to be more prevalent.

The upper age limit of 50 was selected for the following reasons. Based upon the Zurich study, subjects who gained more weight as a result of sleeping shorter hours were in their third and fourth decade. At older ages there is an increase in comorbidity and use of medications, which would introduce a substantial source of variability. The need for sleep changes with age and starting from the fourth decade, changes in sleep architecture take place, which are characterized by increased number and duration of awakenings and by a 60-70% decrease in SWS with concomitant GH insufficiency. Therefore, subjects between the ages of 18 and 50 are more likely to benefit from this intervention, as their physiological needs for sleep are greater.

Only obese subjects (BMI between 29 and 55) who meet the other study criteria will be eligible. Although we believe that chronically sleep deprived subjects with a normal BMI would also benefit from the same intervention, we wish to concentrate on obese subjects who are likely to benefit the most. This inclusion criterion introduces, however, another level of complexity as obese subjects will be more likely to suffer from sleep apnea as well as from all the other medical consequences of obesity (see infra). Subjects will be screened for obstructive sleep apnea by monitoring in their home using the ARES Unicorder (Westbrook 2005), which is a miniaturized monitor worn on the forehead during sleep.

Both sexes will be studied as the prevalence of obesity and sleep deprivation affects both. Since the epidemiology of chronic sleep deprivation teaches us that men and women tend to deprive themselves of adequate sleep to the same extent, we would expect to enroll a similar numbers of men and women. We will exclude menopausal women, as the menopause, which is associated with various non-specific sleep disturbances, including insomnia (usually triggered by night sweats), would increase the variability of the sample [Kristal 1998]. A study of normal menopause transition [McKinlay, 1992] found that the median age of natural menopause was 51.3, so at an upper age limit of 50 we will still potentially be able to include one-half of the applicants.

Naturally “short sleepers” that would be unable to extend sleep will be excluded as they would dilute any beneficial effect of the treatment [Aeschbach 2003]. Although we anticipate that the number of “short sleepers” will be considerably fewer than the sleep-deprived among individuals who sleep less than 6 hours, but that differentiating the two may be difficult, we would identify short sleepers as individuals for whom getting 3-6 hours of sleep a night does not appear to be a problem in that they feel rested and do not experience daytime sleepiness, and do not have a need for napping or catch-up sleep on weekends (Monk 2001).

Subjects whose habitual total daily caffeine intake is more than 500 mg will be excluded because of the potential for interference with sleep [Pollak 2003].

**STUDY IMPLEMENTATION**

(left blank intentionally)


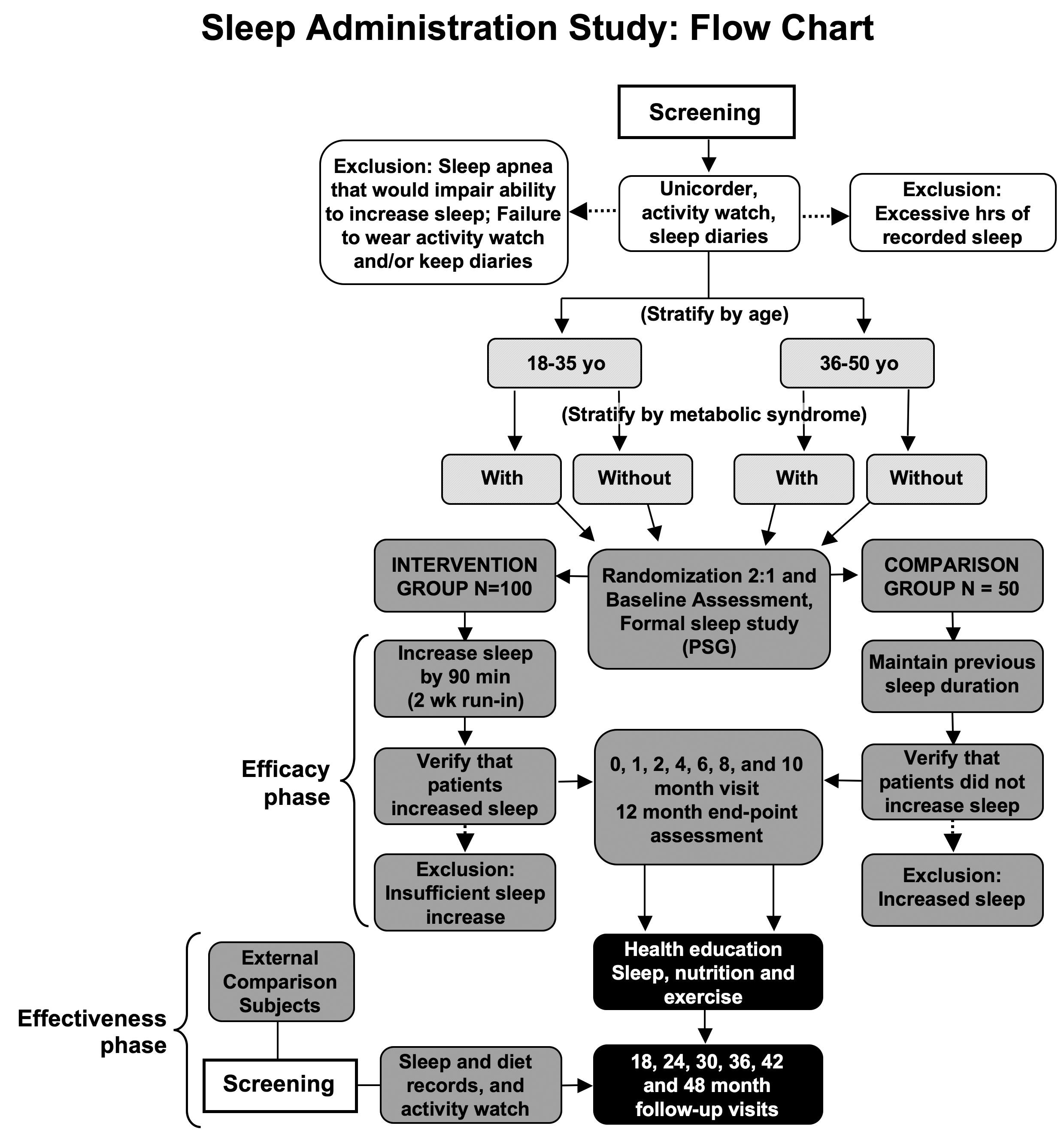


***Standardization and calibration of research procedures***

#### At the beginning of the study the personnel involved in sleep recording will be trained and certified in recording polysomnography data. Recording will be done with PSG equipment on a patient care sleep unit at the Clinical Research Center.

####

#### *Visits*

All visits will take place at the NIH Clinical Research Center.

*Pre-screening phone interview*

A phone interview will assess the subject’s demographics, including age, gender, race, height, and weight; weight loss over the previous 6 months, enrollment in a weight loss program or using active strategies for weight loss, and sleep patterns. Other questions regarding past history of weight changes, current medications, history of diseases, alcohol consumption, and other relevant questions including potential ability to comply with study requirements and plans to move to another geographical area will be asked. Eligible patients will be called in for an in-person screening visit.

*Screening Visit, Outpatient, minus week 5*: The purpose of this visit is to assess whether the subject meets the inclusion/exclusion criteria of the study. During the screening visit, subject’s consent will be obtained; a complete physical will be completed, including measurement of height, weight, and waist, hip, and neck circumference, and a fasting blood sample will be taken and an EKG done. This consent (screening consent) will describe the rationale and general goals of the study, including the expected endocrine changes, but will have little mention of the potential benefits of increasing sleep duration on weight loss. History of medications, current sleep patterns, level of physical activity (retrospective), quality of life, mood, and anxiety will be assessed. Subjects will be asked to fill out the Pittsburgh Sleep Quality Index (PSQI), a self-rated questionnaire, which assesses sleep quality and disturbances [Buysse DJ et al. 1989] and the Owl and Lark Questionnaire [Horne and Ostberg, 1976] to determine morningness-eveningness in circadian rhythms and serve as a guide in coaching for sleep intervention. We will inquire as to the specific reasons that subjects are sleeping 6 ½ hours or less, and verify that modification of behavior will be possible for them. (This information, in addition to identifying good study subjects, will be invaluable in disclosing reasons for the prevalence of inadequate sleep.) In addition, we will provide the potential subjects with an activity watch and a sleep diary, explain the use of the equipment, and inform the subjects about monitoring and maintaining their natural sleep habits. They will be instructed to use the activity watch and maintain a sleep diary for the next two weeks, carefully recording all time asleep, including naps. (For subsequent visits, subjects will be asked to wear an activity watch and fill out the sleep diary for the two weeks preceding the visit.) We will analyze the data and give feedback and evaluate the subject for further participation. Subjects will also be introduced to the exercise diary which they will complete two weeks of each month, and instructed to complete a 3 day food record prior to the next visit. They will be outfitted with and instructed in the use of the Unicorder that they will wear one night while sleeping at home to detect sleep apnea. Female subjects will be given a calendar with instructions to record all menstrual bleeding throughout the study.

Subjects who by this screening have sleep apnea at a level of severity that would likely impair their ability to increase sleep will be excluded from the study. Any subject who is diagnosed with sleep apnea with 20 or more episodes per hour will be advised to seek treatment. After 3 months of treatment and reduction of episodes, they would be invited to reapply for the study. All subjects will receive feedback on their current sleep habits.

Compliance with the activity watch and sleep diary will be assessed with the goal of early identification of poorly compliant subjects and excluding them. (See Study Design and Methods: Study Limitations and Challenges: Compliance with study regimen.) Based upon their willingness and ability to wear the activity watch and regularly fill in the sleep diary to record current sleep patterns, subjects will be invited to proceed with the Randomization visit.

External comparison subjects for the Effectiveness (3 Year Follow-up Phase) study will be screened using identical procedures. At each Main visit (Months 12, 24, and 36 of the extension) a different external comparison will be recruited for the same case and an effort will be made to study external comparisons only once.

*Randomization Visit, Inpatient (two nights), minus week 3*: Subjects remaining in the study at this point will be randomized to the Intervention or Comparison Group. Informed consent for continuation in either the intervention or comparison arm of the study will be obtained. After randomization subjects will have a polysomnography study to collect sleep EEG information, such as the pattern and stages of sleep, with an adaptation night preceding it when the subject is able to stay for two nights.

After randomization to the Intervention or Comparison Group, subjects in the Intervention Group will be coached on increasing sleep over the next two weeks. Detailed instructions on how to increase sleep time, individualized to the subject’s circumstances, will be given to members of the Intervention Group. Additional sleep time will be added in the evening, which is preferable, or early morning. The amount of additional time should be at least 90 minutes or as much as is needed to reach 7.5 hours of sleep (i.e. if a subject randomized to the Intervention Group habitually sleeps 5 hours per night, he/she should sleep between an additional 90 minutes and 2.5 additional hours). Subjects in the Comparison Group will be allowed to maintain their customary sleep habits.

During this visit, baseline endocrine, metabolic and vigilance testing will take place. Fasting blood will be drawn at 8 a.m. for endocrine and metabolic measures and inflammatory markers. Urine will be collected for 24 hours. The visit will include a multiple sleep latency test (MSLT), oral glucose tolerance test (OGTT), indirect calorimetry, and Cooper exercise test or YMCA 3-minute Step Test. The YMCA Step Test [Hong, 2000] is a less strenuous measure of fitness and will be used for those subjects who cannot perform the Cooper 12 minute walk/run test. Subjects will perform the Psychomotor Vigilance Task, a measure of reaction time to gauge alertness/sleepiness. This will be done at each subsequent visit. Sleep diaries and exercise records for the past two weeks will be collected and evaluated. Measurements of height, weight, waist, hip, and neck circumference will be obtained. Appetite, sleepiness, quality of life, personality, mood, and anxiety will be assessed and a screening for chronic pain syndromes will be done. Neuropsychological testing will be performed. A structured interview (SCID) for DSM-IV diagnoses and personality testing will be done. Subjects will be asked to rate their level of sexual satisfaction on a visual analog scale. Subjects will receive further instruction as needed for maintaining exercise and sleep diaries for the two weeks preceding each subsequent visit, their 3-day food record will be collected. They will be instructed to wear the activity watch before the next visit. They will also be introduced to the activity monitor (Actical) that they will wear at the waist to record amount and levels of physical activity for two weeks preceding the next visit, and each subsequent visit, along with the activity watch (Actiwatch). Each subject will be given a scale and a blood pressure monitor and instructed to obtain measurements at a prescribed time. Their measurements will be compared to values obtained at the visit to serve as validation for the interim measurements at months 3, 5, 7, 9, and 11.

*0 Month Visit, Outpatient, time 0:* We will assess subject compliance towards study requirements in both groups. Those subjects who successfully comply with study requirements of increasing sleep (Intervention Group) or maintaining habitual sleep routine (Comparison Group) will continue with the study. (See Study Design and Methods: Study Limitations and Challenges: Compliance with the study regimen.) During the run-in phase participants should have recorded a two-week diary of sleep time, increased sleep duration by an average of 30 or more minutes/night (Intervention Group) or not increased their sleep duration (Comparison Group). (However, up to 30 minutes increase will be tolerated in the Comparison Group.) Those subjects who did not comply with the instructions within their group may be excluded.) Fasting blood will be drawn for endocrine and metabolic measures, appetite and sleepiness assessed, and vigilance testing done. At this visit the baseline measures of a DEXA scan of body composition, CT scan of abdominal fat, plethysmography (BOD POD), and bioelectric impedance will also be done. The subjects will wear the activity watch and complete sleep diaries for 2 weeks before the next visit, and each subject will be issued a pedometer to be used throughout the study as an additional measure of physical activity.

*Interim Visits, Outpatient, months 1, 2, 4, 6, 8, and 10:* Subject’s weight will be recorded. Any problems associated with the study will be addressed, and importance of compliance will be reinforced. Sleep and exercise diaries for the past two weeks will be evaluated, and exercise recorded. and the subject will do a 24-hour recall of food eaten. Sleepiness and appetite will be assessed. Mood and anxiety ratings may be done. A fasting blood sample will be obtained for endocrine and metabolic measures. Actiwatch and Actical data will be collected; and the subject’s own measures of weight and BP will be compared with investigator’s measures for validation. For the 4-month visit, subjects will complete and bring 3-day food records to be analyzed.

*End-point Visit of Randomized Phase, Inpatient (two nights), month 12:* An inpatient stay similar to the one at the baseline assessment visit at Randomization will occur at month 12. In order to repeat the polysomnography and allow an adaptation night it may be a 2-night stay. During this inpatient visit, end-point endocrine, metabolic and vigilance testing will take place, and procedures from the Randomization and 0 month visits will be repeated. During this visit all subjects (both Comparison and Intervention Group) will take part in a health education session in which we will discuss the potential benefits of adequate sleep and encourage increased sleep. We will also present information about nutrition and exercise for a healthy lifestyle. This visit will conclude the intervention part (Efficacy [Randomized] Phase) of the study.

*Follow-up Visits, Outpatient and Inpatient, months 18, 24, 30, 36, 42, and 48:* In the post-treatment “effectiveness” visits all subjects (both in the Intervention and Comparison Groups) will be re-evaluated at 6 month intervals after the termination of the trial to evaluate the impact of healthy sleep habits, proper diet, and exercise in a “real life situation,” and to ascertain if changes can readily be made and become effective even without an intensive year of effecting change in sleep. In addition, these factors will reveal whether new discoveries can translate into stable life style changes. For subjects who consent to the Effectiveness (3 Year Follow-up) phase, there will be outpatient (Interim) visits at 18, 30, and 42 months, and inpatient (Main) visits at 24, 36, and 48 months from their initial enrollment. For the duration of the Effectiveness (3 Year Follow-up) Study Phase, subjects will wear an activity watch and monitor and maintain sleep diaries for one week of each month, during which week they will record weight and blood pressure once, and pedometer steps daily.

*Interim visits (18, 30, and 42 months)*: At these outpatient visits, sleepiness, appetite, and activity will be evaluated; weight, blood pressure, waist, and neck measurements will be obtained; mood and anxiety will be rated; and fasting blood samples will be obtained for endocrine and metabolic measures. The healthy lifestyle plan (sleep, diet, exercise) developed with the subject at the 12-month visit will be reviewed.

*Main visits (24, 36, and 48 months):* These will be inpatient visits of one to three nights, in which all of the evaluations done at the interim visits will be repeated, and additional testing done. Quality of life, sexual satisfaction, and pain will be assessed, and a psychological interview (SCID) will be conducted. Three-day food records will be collected and analyzed. Indirect calorimetry for resting energy expenditure will be done, and an oral glucose tolerance test will be performed. Body composition will be measured by DEXA scan, bioelectric impedance, and Bod Pod, and abdominal fat will be measured by a CT scan. Polysomnography and a multiple sleep latency test may be included. Neuropsychological testing will be repeated.

A 24-hour urine collection will be obtained, and blood will also be drawn for ACTH and cortisol, TSH and FT4, GH and IGF-1, testosterone, and inflammatory markers. Again, the healthy lifestyle plan for sleep, diet, and exercise will be reviewed, with encouragement and additional information as needed.

The Main Visits of the Effectiveness (3 Year Follow-up Phase) study are the only visits in which the external comparison subjects will participate. They will be screened and matched individually to an enrolled subject (case) to serve as a comparison for preferably a single visit. At the visit, they will undergo the same procedures, but will not be exposed to the healthy lifestyle information.

***Subject withdrawal***

If a subject indicates that they do not wish to continue in the study but is amenable, a final visit will be done to collect end-point data. If the subject is in the Efficacy (Randomized) Phase and at least 3 months past Baseline, a “12 month” visit would be done at that time. (DEXA and CT would not be done unless the subject were at least 6 months past Baseline). If the subject were at least 3 months into the Effectiveness (3 Year Follow-up) Phase, an “18 month” visit would be done.

***Phone contact***

All subjects will be called between visits for purposes of addressing concerns, problem solving, reminders to complete diaries and measures, and confirmation of visit appointments. In addition they will all be provided with the phone contact numbers of the study team.

**Schematic of Study Visits**

| VISIT | | PROCEDURES | **TIMELINE** |
| --- | --- | --- | --- |
| Screening  (Outpatient) | | Informed consent  Explanation of study rationale and goals  History and physical examination  Measurements of blood pressure, height, weight, waist, and neck and hip circumference  Electrocardiogram  Fasting blood sample  Sleep questionnaires  AAFQ (Activity questionnaire)  Sleepiness and appetite assessment  Quality of life, mood and anxiety assessment  Retrospective assessment of physical activity  Introduction of exercise diaries  Introduction to activity watch and sleep diaries  Instruction for 3 day food record for next visit  Provision of Unicorder to wear at night at home  Women start recording menstrual bleeding | -5 weeks |
| Randomization  Baseline Assessment  (Inpatient, 1-2  nights) | | Evaluation of compliance with sleep and exercise diaries and activity watch  Inclusion or exclusion  Randomization to intervention or comparison group  Informed consent  Polysomnography for sleep EEG  Instruction (coaching) for sleep trial  Fasting blood sample for baseline metabolic, endocrine and inflammatory marker assessmentSleepiness and appetite assessmentPVT (psychomotor vigilance task)  AAFQ (Activity questionnaire)  Measurements (bp,wt.waist, neck) Sleepiness and appetite assessment  Collection of 3 day food record  Personality testing  Provision of scale, blood pressure monitor  MSLT  GTT,  Indirect calorimetry  Cooper test or Step test  Quality of life, mood and anxiety assessment  Screening for chronic pain syndromes  Neuropsychological testing  Instruction re waist activity monitor (Actical) | -3 weeks |
| 0 Month Visit  (Outpatient) | | Evaluation of compliance with sleep prescription (increase for intervention group; maintenance of habitual routine for comparison group) and use of sleep diary  Inclusion or exclusion  Fasting blood sample (hormones, glucose, lipids)  Measurements (ht. bp., wt., waist, hip, neck circumference)  Exercise diary collection and evaluation  AAFQ (Activity questionnaire)  24 hour food recall  Collection of activity watch and activity monitor data  Sleepiness and appetite assessment  PVT (psychomotor vigilance task)  SCID  Mood and anxiety ratings  DEXA, and CT  BOD POD  Bioelectric impedance  Pedometer given | Time 0 |
| Interim Visits  (Outpatient) | | Collection of activity watch and activity monitor data  Collection and evaluation of sleep and exercise diaries  24 hour food recall  3 day food record at month 4  Measurements (bp, wt., waist, neck)  Validation of subject self-measurement; recording of months previous  Fasting blood sample (hormones, lipids, glucose)  AAFQ (Activity questionnaire)  Sleepiness and appetite assessment  PVT (Psychomotor vigilance task)  Mood and anxiety ratings | Months 1, 2, 4, 6, 8, and 10 |
| End-point Assessment Visit  (Inpatient, 1-2 nights) | | (Repeat of Randomization visit)  Fasting blood sample for end-point metabolic, endocrine  and inflammatory marker assessment  Measurements (bp, wt., waist, hip, neck)  Collection of activity watch and activity monitor data  Exercise and sleep diary collection and evaluation  PVT  24 hour food recall  Collection of 3 day food record  Record self-measured weight and BP for month11.  Sleepiness and appetite assessment  AAFQ (Activity questionnaire)  PSG plus MSLT  GTT  DEXA and CT  BOD POD  Bioelectric impedance  Indirect calorimetry  Cooper test or step test  Quality of life, mood and anxiety assessment  Screening for chronic pain syndromes  Health education session with information on exercise, nutrition, and sleep  **Unicorder**  **End of efficacy (randomized) phase of study** | Month 12 |
| Follow-up Visits | Interim  (Outpatient) | Measurements (weight. waist, bp, neck)  Collection of subject self-measurements (weight, bp)  Fasting blood sample (hormones, glucose, lipids)  Sleepiness and appetite assessment  Activity questionnaire  Mood and anxiety rating  Review of healthy lifestyle plan | Months 18, 30, 42 |
|  | Main  (Inpatient, 1-3 nights) | Measurements (weight., waist, neck, bp)  Fasting blood sample  Sleepiness and appetite assessment  Activity questionnaire  Review of 3-day food record  PSG plus MSLT  Bioelectric impedance  Quality of life, pain, mood and anxiety assessment  SCID  DEXA. Bod Pod, and BIA for body composition  CT scan (abdominal fat)  OGTT  Neuropsychological testing  24 hour urine collection  **End of effectiveness (3 year follow-up) phase of study** | Months 24, 36, 48 |

**ANALYSIS OF THE STUDY**

*Stratification*

Subjects will be stratified based upon two factors: age and the presence of metabolic syndrome.

*Metabolic syndrome:* At baseline, we will assess the distribution in the number of components of the metabolic syndrome in study participants. The main analysis will categorize study participants as having the metabolic syndrome (3 or more components) or not having the metabolic syndrome (less than 3 components). Although we cannot predict the exact prevalence of metabolic syndrome in our sample, we will enrich our sample with subjects with the metabolic syndrome by making sure that at least 50% of our subjects will have metabolic syndrome. A secondary analysis will establish the percentage of individuals who have 0, 1, or 2 components of the criteria that make up metabolic syndrome, and therefore do not meet full requirements of the metabolic syndrome, or at the other extreme, have 3, 4, or 5 components of the syndrome. This additional analysis should increase the sensitivity of the study to detect potential changes in the components of the metabolic syndrome, as a result of the sleep intervention. In addition, changes in the values of each component (blood pressure, total cholesterol, HDL, waist circumference, triglycerides) between baseline and month 12 will be also analyzed as continuous variables. Similar weight will be assigned to each clinical component of the metabolic syndrome.

*Age:* Subjects will be stratified according to two age groups; approximately 50% of our subjects will be between the ages of 18 to 35, and the other half between the ages of 36 to 50.

There will be blocked randomization for these two factors (age, and presence/absence of metabolic syndrome) to ensure that a similar proportion of subjects with metabolic syndrome are randomized to both the intervention and comparison group, and a similar proportion of younger subjects will be randomized to each group. After randomization, the presence of significant differences in age and presence of metabolic syndrome will be assessed by means of paired t test or Wilcoxon test. The study hypothesis is that an increase in sleep time will be associated with a decreased prevalence of metabolic syndrome, and/or with a decrease in the number of individual components of the metabolic syndrome. Based upon this hypothesis, changes in sleep time (expressed in minutes) will be inversely related to the presence/absence of metabolic syndrome and/or to individual components of it. Caloric intake and amount of physical activity will be covariates.

Another important variable of study will be the number of hours slept at study entry. For example, it is likely that there will be fewer subjects who sleep 4 hours or less (arbitrarily defined here as severely sleep deprived) than subjects who sleep 6 hours (mildly sleep deprived). Since it would not be feasible to stratify also for this third variable, at the time of analyzing the data, we will adjust our analysis for this variable (average number of hours slept at study entry).

2:1 *Allocation*: in order to gather more information on the effects of this novel non-pharmacological intervention, sleep extension, we propose a 2:1 allocation ratio between the Intervention and the Comparison Groups. It is possible however that during the execution of the study, the targeted 2:1 allocation ratio allocation may change because of more subjects dropping out or being excluded from one or the other study groups. In case we notice a trend towards a substantial deviation (i.e. 3:1 or more) from the allocation originally intended, we will randomize additional patients by using a different allocation ratio aimed at correcting the imbalance, a process usually referred to as “adaptive randomization”.

*Sample Size Determination*

*Changes in BMI.* Hasler et al. [2004] reported the average annual change in body mass index (BMI) by the average sleep duration in hours. The group with the least number of sleep hours (less than 5 per night) had a yearly increase in BMI of approximately 0.38 on average with a standard deviation of 0.35. These numbers are based on a longitudinal follow up study of nearly 600 people [Hasler et al., 2004]. The present 12 month comparison- controlled randomized intervention trial of subjects who chronically sleep less than 6 hours proposes an additional 90 minutes of sleep at night (intervention) compared to a continuation of a habitual short sleep schedule (comparison). Change in BMI between baseline and 12 months will be compared between those who get an additional 90 minutes of sleep versus those who do not.

Table 1. Sample size needed for detecting changes in BMI as a result of 12 months of sleep administration. All calculations are based upon two-sided t test.

| Scenario | ∆ in BMI between the 2 groups at the end of the 12 month intervention period  (unit of BMI) | N of subjects/group  With 80% power  (P=0.05 two sides) |
| --- | --- | --- |
| No increase in BMI in the intervention group | 0.38±0.35 | 15 |
| Increase in BMI is reduced by one half in the intervention group | 0.19±0.35 | 54 |

Two possible scenarios are contemplated in the table. Both scenarios are based upon the assumption that subjects in the comparison group experience an increase in BMI over 12 months identical to what is reported by Hasler et al. ((i.e. 0.38 kg/m^2^ of BMI on average with a standard deviation of 0.35 (increase in BMI of 0.38±0.35)). This is a quite conservative assumption as the sample studied by Hasler was different in that it was comprised of mostly non-obese subjects. It is reasonable to assume that the annualized rate of BMI increase will be higher in our study, as our subjects will all be obese. In addition, the annualized rate of BMI increase in the general population is likely to be greater in the US than in Switzerland.

In the first scenario, our intervention group would not experience an increase in BMI over 12 months. As reasons for BMI increases are likely to be various, we have also contemplated a second scenario in which the annual increase in BMI in the Intervention Group would be reduced only by one half. In both cases there is sufficient power for a proof-of-concept intervention study (80%) and more than what is usually required in terms of statistical power (P = 0.05) for these kinds of exploratory studies. Even if our intervention is effective in cutting only in half the annual increase in BMI, thus preventing a 0.19 increase in BMI/year, this is equivalent to saving approximately one pound/yr. Over a 10 year time-period this would translate into a “savings” of about 10 pounds, making this sleep extension intervention clinically meaningful .

Based upon the above, we plan to enroll 100 subjects in the Intervention Group and 50 subjects in the Comparison Group in order to complete the 12-month intervention with a sufficient number of subjects in each group. This would allow for a 30 % dropout rate in each group while retaining sufficient power. It is estimated that it will be necessary to prescreen at least twice (approximately 300 subjects) the number of subjects than is needed. It should be noted that this analysis is very conservative as it was based upon a sample with different characteristics, i.e. subjects with normal sleep and normal BMI. Based upon two other studies, the study by Kripke and the study by Mignot [Kripke 2002 and Taheri 2004] which recruited subjects with demographic characteristics more similar to the sample of this protocol, we predict that the difference in BMI observed as result of the treatment will be greater, probably 0.50 unit of BMI, in which case even with 9 subjects/group there would be sufficient power to detect a difference. Efficacy (Randomized Phase) data will be assessed by an intention-to-treat analysis, which will include all subjects who were randomized and had at least one efficacy measurement (either at Month 2, 4, 6, 8, or 10). The analysis involving changes from baseline will use the last observation carried forward by fitting a linear regression to the data and using the slope.

*Changes in ghrelin and leptin*: The secondary hypothesis is that sleep administration will induce changes in ghrelin and leptin of opposite direction and similar magnitude to the changes observed as result of sleep deprivation, which were characterized by an increase in ghrelin and a decrease in leptin. Therefore, we assume that sleep administration will result in an increase in plasma leptin and a decrease in plasma ghrelin.

For the purpose of performing a power analysis, fasting ghrelin levels were obtained from the literature in a subject sample very similar to ours [McLaughlin et al. 2004]. In this sample with similar characteristics to our study subjects, consisting of 20 obese men and women with insulin resistance, ghrelin levels were 252+85 pg/ml (mean+SD). The increases reported in the orexogenic peptide ghrelin, as result of sleep deprivation ranged from 28% in the Van Cauter study to 15% in the Mignot study. To be conservative, we will assume that paying the sleep debt will result in a 15% decrease in fasting ghrelin levels (therefore from 252 to 214 pg/ml mean); we assume that the SD will remain similar (85) (other relevant parameters P = 0.05; power 0.8 (table); to allow for missing data).

Table 2. Sample size needed for detecting various changes (decrease) in fasting ghrelin (SD = 85), as result of 12 months of sleep administration. All calculations are based upon two-sided t test. Mean+SD are from McLaughlin et al. 2004.

|  | P= 0.05;  power 0.80 | P= 0.05;  power 0.90 | P= 0.1;  power 0.80 | P= 0.1;  power 0.90 |
| --- | --- | --- | --- | --- |
| ghrelin  (15% decrease to 214 pg/ml) | N=80/group | N= 106/group | N=62/group | N=86/group |
| ghrelin  (20% decrease to 202 pg/ml) | N=46/group | N= 61/group | N=36/group | N=50/group |
| ghrelin  (28% decrease to 181 pg/ml) | N=23/group | N=31/group | N=18/group | N=25/group |

In addition the study will have ample power to detect increases in leptin of larger magnitude than the changes already reported in the literature.

Table 3. Sample size needed for detecting various increases in fasting leptin (35.6+4.2 mean+SD in a sample of 22 obese women), as a result of 12 months of sleep administration. All calculations are based upon two-sided t test. Mean+SD are from Guven S. et al. 1999.

|  | P= 0.05;  power 0.80 | P= 0.05;  power 0.90 | P= 0.1;  power 0.80 | P= 0.1;  power 0.90 |
| --- | --- | --- | --- | --- |
| leptin  (10% increase to 39.16 ng/ml) | N=23/group | N=31 /group | N=18/group | N= 25/group |
| (15% increase to 40.94 ng/ml) | N= 10/group | N=14/group | N=9/group | N=11/group |
| (20% increase to 42.72 ng/ml) | N=7/group | N=8/group | N=5/group | N=7/group |

## Data Safety Monitoring Plan

## An independent panel of three physicians (two experts in the fields of endocrinology and one in the field of sleep research) will constitute a data safety monitoring board. This board will meet on a routine schedule every 3 months to review patient data as well as at unscheduled times according to clinical necessity (e.g. to review (severe) adverse events or discuss major medical decisions).

## Adverse Events Reporting

All adverse events will be reviewed weekly. All serious adverse experiences will be reviewed within 24 hours. A report will be forwarded to the NIDDK IRB with a copy to the Clinical Director, as soon as possible, but no later than seven (7) days in the case of death or life-threatening serious adverse events or within fifteen (15) days after the occurrence of all other forms of serious adverse events.

**HUMAN SUBJECTS PROTECTIONS**

## Recruitment

Given the current awareness of obesity and the growing awareness of the negative effects of sleep deprivation, we expect that the community will be primed to the issues that this study addresses. We will advertise through radio and printed journals to attract obese (BMI between 29 and 55) subjects who sleep less than six hours during the week, wake up feeling tired, and feel that they need more sleep. Subjects will be recruited from the community in the Washington-Baltimore area. Participation of minorities and subjects of low socioeconomic status will be strongly encouraged, given also the epidemiology of sleep deprivation and obesity. Special collaborative agreements may be put in place with local research centers to facilitate recruitment of this segment of the population.

*Compensation*

Subjects will receive reimbursement for the time they spend in the hospital for inpatient or outpatient visits, as well as for the inconvenience of the procedures they undergo. In addition, a small monthly stipend will be given to participants in both the intervention and comparison groups to compensate them for the inconvenience associated with recording sleep patterns and food intake, and for changing their usual sleep patterns. All subjects will be given a scale, pedometer, and blood pressure monitor for use during the study, which they will be allowed to keep at the end of the study. A complete schedule of compensation is included in Appendix 4.

*Benefits*

Subjects will receive the benefits of medical evaluation, dietary evaluation, polysomnography, and education about proper nutrition, adequate exercise, and sufficient sleep for a healthy lifestyle.

### Risks and discomforts

Since there are no experimental drugs involved in the protocol, the potential for risk is reduced. Radiation risks are associated with CT and DEXA. The effective dose for both procedures is of minimal risk. The Cooper test entails the risks associated with exercise (abnormal blood pressure or heart rate, or in rare instances, heart attack). However, the risk is statistically small (a fatality rate of 1 death per 1.51 million adult exercise tests has been reported). An alternative test of fitness, the YMCA 3-minute Step Test, will be used for those subjects for whom the Cooper test might be too strenuous. Venipuncture for blood samples carries a very small risk of infection at the site. Other discomforts encountered in the study include possible muscle or joint soreness following exercise, the discomfort and possible bruising from blood draws, and restriction of movement, discomfort, and possible skin irritation from the electrodes and other apparatus applied for the polysomnography. There may be inconvenience associated with the time required to fill out questionnaires and logs, and with wearing the activity watch. Wearing the activity watch may occasionally result in a temporary discoloration of the skin or a mild irritation, which resolves when the watch is removed. Wearing the Unicorder (which is worn strapped to the subject’s forehead) may leave a reddened area of the skin, and infrequently may result in a skin eruption that may cause mild discomfort but resolves spontaneously without pharmacological intervention.

For specific categories of workers, such as cab, bus, and truck drivers, pilots, and heavy machinery operators, for whom sleepiness may be especially hazardous, this study might present a risk greater than for others because they might be randomized to the comparison group and not increase sleep. Therefore, they may not be eligible to participate. In addition, the consent form will disclose the potential risk of accidents due to sleepiness that may ensue in the course of the study even though it does not directly result from the study.

**RESEARCH USE AND DISPOSITION OF HUMAN SAMPLES AND DATA**

Human samples collected during the course of the study (blood) will be stored in freezers in the NIH laboratories indefinitely for future analysis related to obesity and sleep. All samples will be identified by a study code linked to patient name and identification number. Individual subjects will not be identifiable in the results of any analysis.

Human data that is collected, such as physiological measures (energy expenditure, physical activity, food intake, abdominal fat, body composition, and polysomnography), neuropsychological tests, and mood, anxiety and symptom ratings will be stored indefinitely in the medical record and other secured databases at NIH.

Any human samples or data that may be sent away from NIH for analysis will be coded so that individual subjects cannot be identified. Frozen serum from blood samples may also be shipped to the labs of Drs. Allison, Dhurandhar and Atkinson to be analyzed for adenovirus and other possible infective causes of obesity. The Arizona Activity Frequency Questionnaires (AAFQ) will be mailed to the University of Arizona (Ellen Graver, The Arizona Diet and Behavioral Assessment Center) where they will be scored and analyzed and an electronic file of energy expenditure results returned. The recordings from the Unicorder will be transmitted electronically to the company that makes the device, Advanced Brain Monitoring, for editing and production of a final report (under the direction of Dr. Delmer Henninger) on the parameters of sleep and respiratory disturbance to screen for sleep apnea. The samples and data that are sent will not have subjects’ names or identifying information, but will be coded with a subject number that only the investigators at NIH will be able to connect with the subject.

Maintaining codes for human biological samples as well as research data is justified by the need to identify individual research data as well as individual biological samples. Please note that no obvious identifier such as subject initials or date of birth will be placed on the label.

In the unlikely event that an accidental loss or unintended destruction of a sample will take place, the principal investigator will promptly report such incident to the IRB. Please note that for sample collection and storage we follow rigorous standards of practice, which are being implemented by the NIDDK Core Biological Sample Lab Team. This group is specifically charged with the task of promptly collecting, processing, aliquotting, labeling and inventorying the samples, and with retrieving samples for pre-specified analysis only upon the specific request of the PI.

**STUDY PUBLICATIONS**

*Rationale*

Because of the interdisciplinary nature of this study, several NIH institutes as well as non-NIH academic institutions have worked together in a very collegial atmosphere in the preparation of this protocol. We hope that this atmosphere will continue, as it would greatly contribute to the success of this study. To foster and maintain such a collegial climate, which is conducive to high scientific productivity, a Publication and Data Analysis Committee for this study will be created. The following guidelines, although the sole responsibility of the Sleep Administration Study Team, are modeled after the Publications and Presentations Guidelines of the Health ABC Study, another NIH interdisciplinary study [Harris T. et al, Health ABC Publications and Presentation Guidelines, NIH 4/21/2000 Society for Neuroscience. Responsible conduct regarding scientific communication. First Edition 1998] and the Authorship Guidelines of the Harvard Medical School [1996 <http://www.hms.harvard.edu/integrity/authorship.html>].

#### Official study name

The official name of the study, for scientific purposes, is the Sleep Administration Study. This name should be used in all publications and presentations.

**Functions of the Publication and Data Analysis Committee**

The main goals of the Publication and Data Analysis Committee will be:

- To encourage high quality publications and presentations produced in a timely fashion.
- To encourage broad participation by Study Investigators in publications and presentations.
- To ensure fair authorship according to the International Committee of Medical Journal Editor Guidelines.
- To provide a forum in which decision upon authorship and other related issues are openly made by a committee legitimized by all investigators.

## **Authorship**

Authors should participate in the writing of the paper in accordance with the International Committee of Medical Journal Editors guidelines. Authorship is an explicit way of assigning responsibility and giving credit for intellectual work. The two are linked. Authorship practices should be judged by how honestly they reflect actual contribution to the final product. Authorship is important to the reputation and grant support of the individuals involved as well as to the strength and reputation of their institutions.

- Everyone who is listed as an author should have made a substantial, direct, intellectual contribution to the work. For example, in the case of a Sleep Administration Study research report, they should have contributed to the conception, design, analysis and/or interpretation of data.
- Acquisition of funding and provision of technical services, patients, or materials, while they may be essential to the work, are not in themselves sufficient contributions to justify authorship.
- Everyone who has made substantial intellectual contributions to the work should be an author. Everyone who has made other substantial contributions should be acknowledged.
- When research is done, as in the case of the Sleep Administration Study, by teams whose members are highly specialized, an individual’s contributions and responsibility may be limited to specific aspects of the work.
- All authors should participate in writing the manuscript by reviewing drafts and approving the final version.

One author should take primary responsibility for the work. This primary author should assure that all authors meet basic standards for authorship and should prepare a concise, written description of their contribution to the work, which has been approved by all authors. This record should remain with the sponsoring department.

*Order of authorship*

- The authors should decide the order of authorship together.
- Authors should specify in their manuscript a description of the contribution of each author and how they have assigned the order in which they are listed so that the readers can interpret their roles correctly.
- The primary author should prepare a concise, written description of how order of authorship was decided.

ACKNOWLEDGEMENTS

We would like to thank the individuals who provided constructive insight to the development of this protocol. A one-day investigators meeting was held at the NIH CC on January 21, 2005 to discuss the protocol and the following were invited:

Ann Berger, Karim Calis, Giovanni Cizza, George Csako, Andrea Deak, Patricia Deuster, Bart Drinkard, Wallace Duncan, Janet Gershengorn, Gregor Hasler, Mac Donald Horne, Carl Hunt, Saul Malozowsky, Husseini Manji, Merrill Mitler, Thomas Mellman, Kathleen Merikangas, Emanuel Mignot, Terry Phillips, Frank Pierce, Norman Rosenthal, David Rubinow, Susumi Sato, Nancy Sebring, Monica Skarulis, Nina Sonbolian, Esther Sternberg, Sara Torvik, Tom Wehr, and Bob Wesley.

We thank Nina Sonbolian for her work in preparation and careful editing of the protocol.

**REFERENCES**

Aeschbach D, Cajochen C, Landolt H, Borbely AA. Homeostatic sleep regulation in habitual short sleepers and long sleepers. Am J Physiol. 1996; 270(1 Pt 2):R41-53.

Aeschbach D, Sher L, Postolache TT, Matthews JR, Jackson MA, Wehr TA.

A longer biological night in long sleepers than in short sleepers. J Clin Endocrinol Metab. 2003; 88(1):26-30.

Arnedt JT, Wilde GJ, Munt PW, MacLean AW. How do prolonged wakefulness and alcohol compare in the decrements they produce on a simulated driving task? Accid Anal Prev. 2001;33(3):337-44.

Ayas NT, White DP, Al-Delaimy WK, Manson JE, Stampfer MJ, Speizer FE, Patel S, Hu FB. A prospective study of self-reported sleep duration and incident diabetes in women.

Diabetes Care. 2003; 26(2):380-4.

Ayas NT, White DP, Manson JE, Stampfer MJ, Speizer FE, Malhotra A, Hu FB.

A prospective study of sleep duration and coronary heart disease in women. Arch Intern Med. 2003; 27;163(2):205-9.

Bao G, Guilleminault C. Upper airway resistance syndrome—one decade later. Curr Opin Pulm Med. 2004; 10(6):461-7.

Benca RM, Quintas J. Sleep and host defenses: a review.

Sleep. 1997; 20:1027-37.

Bergner M, Bobbitt RA, Carter WB, Gilson BS. The Sickness Impact Profile: development and final revision of a health status measure. Med Care. 1981;19(8):787-805.

Bonnet MH, Arand DL. We are chronically sleep deprived. Sleep. 1995;18(10):908-911.

Bray GA. Medical consequences of obesity. J Clin Endocrinol Metab. 2004 Jun;89(6):2583-9.

Buysse DJ, Reynolds CF 3rd, Monk TH, Berman SR, Kupfer DJ. The Pittsburgh Sleep Quality Index: a new instrument for psychiatric practice and research. Psychiatry Res. 1989;28(2):193-213.

Castle PE, Phillips TM, Hildesheim A, Herrero R, Bratti MC, Rodriguez AC, Morera LA, Pfeiffer R, Hutchinson ML, Pinto LA, Schiffman M. Immune profiling of plasma and cervical secretions using recycling immunoaffinity chromatography.

Cancer Epidemiol Biomarkers Prev. 2003;12:1449-56.

Centers for Disease Control and Prevention. Overweight and obesity: obesity trends. Accessed at: <http://www.cdc.gov/nccdphp/dnpa/obesity/trend/index.htm> on 27 April 2004.

Cirelli C, Bushey D, Hill S, Huber R, Kreber R, Ganetzky B, Tononi G. Reduced sleep in Drosophila Shaker mutants.

Nature. 2005 Apr 28;434(7037):1087-92.

Cizza G, Romagni P, Lotsikas A, Lam G, Rosenthal NE, Chrousos GP. Plasma leptin in men and women with seasonal affective disorder and in healthy matched controls.

Horm Metab Res. 2005;37(1):45-8.

Cook W, Medley D. Proposed hostility and pharisaic-virtue scales for the MMPI. J Appl Psychol. 1954;38:414-18.

Dawson D, Reid K. Fatigue, alcohol and performance impairment. Nature. 1997 17;388(6639):235.

Di Lorenzo, L., G. De Perloga, et al. Effect of shift worker on body mass index: results of a study performed in 319 glucose-tolerant men working in a Southern Italian industry. Int J Obes Relat Metab Disord 27(11): 1358-8.

Dinges, DF. Sleep debt and scientific evidence. Sleep. 2004; 27(6):1050-2,

Endicott, J., Nee, J., Haarrison, W., Blumenthal, R. Quality of life enjoyment and satisfaction questionnaire: a new measure. Psychopharmacol Bull. 1993;29(2):321-6.

Facchini FS, Hua N, Abbasi F, Reaven GM. Insulin resistance as a predictor of age

related diseases. J Clin Endocrinol Metab 2001; 86:3574-8.

James E. Gangwisch,; Dolores Malaspina, MD, MPH^2^; Bernadette Boden-Albala, Dr.PH^3^;Steven B. Heymsfield, Inadequate Sleep as a Risk Factor for Obesity: Analyses of the NHANES I Sleep *in Press 2005*

Gottlieb DJ, DeStefano AL, Foley DJ, Mignot E, Redline S, Givelber RJ, Young T.

APOE[epsilon]4 is associated with obstructive sleep apnea/hypopnea: The Sleep Heart Health

Study. Neurology. 2004; 63(4):664-8.

Grandner MA, Kripke DF. Self-reported sleep complaints with long and short sleep: a nationally representative sample. Psychosom Med. 2004;66(2):239-41.

Grundy SM, Pasternak R, Greenland P, Smith S, Jr., Fuster V. Assessment of cardiovascular risk by use of multiple-risk-factor assessment equations: a statement for healthcare professionals from the American Heart Association and the American College of Cardiology. Circulation 1999; 100:1481-92.

Guven S, El-Bershawi A, Sonnenberg GE, Wilson CR, Hoffmann RG, Krakower GR, Kissebah AH. Plasma leptin and insulin levels in weight-reduced obese women with normal body mass index: relationships with body composition and insulin. Diabetes. 1999 Feb;48(2):347-52.

Halbach MM, Spann CO, Egan G. Effect of sleep deprivation on medical resident and student cognitive function: A prospective study. Am J Obstet Gynecol. 2003; 88(5):1198-201.

Hartmann E, Baekeland F, Zwilling GR. Psychological differences between long and short sleepers. Arch Gen Psychiatry. 1972;26(5):463-8.

Hasler G, Buysse DJ, Klaghofer R, Gamma A, Ajdacic V, Eich D, Rossler W, Angst J. The association between short sleep duration and obesity in young adults: a 13-year prospective study. Sleep. 2004; 15;27(4):661-6.

Hasler G, Pine DS, Gamma A, Milos G, Ajdacic V, Eich D, Rossler W, Angst J. The associations between psychopathology and being overweight: a 20-year prospective study.

Psychol Med. 2004 Aug;34(6):1047-57.

Hong Y, Li, JX, Robinson, PD. Balance control, flexibility, and cardiorespiratory fitness among older Tai Chi practitioners. Br. J. Sports Med. 2000; 34:29-34.

Horne JA, Ostberg, O. A self-assessment questionnaire to determine morningness-eveningness in hman circadian rhythms. International Journal of Chronobiology. 1976, 4: 97-110.

Irwin M. Effects of sleep and sleep loss on immunity and cytokines.

Brain Behav Immun. 2002;16:503-12.

Jean-Louis G, Kripke DF, Ancoli-Israel S. Sleep and quality of well-being. Sleep. 2000 15;23(8):1115-21.

Kohsaka M, Fukuda N, Honma K, Honma S, Morita N. Seasonality in human sleep. Experientia. 1992; 15;48(3):231-3.

Kripke DF, Garfinkel L, Wingard DL, Klauber MR, Marler MR. Mortality associated with sleep duration and insomnia. Arch Gen Psychiatry. 2002 ;59(2):131-6.

Krystal AD, Edinger J, Wohlgemuth W, Marsh GR. Sleep in peri-menopausal and post-menopausal women. Sleep Med Rev. 1998;2(4):243-53.

Landrigan CP, Rothschild JM, Cronin JW, Kaushal R, Burdick E, Katz JT, Lilly CM, Stone PH, Lockley SW, Bates DW, Czeisler CA. Effect of reducing interns' work hours on serious medical errors in intensive care units. N Engl J Med. 2004; 28;351(18):1838-48.

McKinlay, SM, Brambilla, DJ, Posner, JG. The normal menopause transition. Maturitas. 1992; 14(2): 103-15.

McLaughlin T, Abbasi F, Lamendola C, Frayo RS, Cummings DE..Plasma ghrelin concentrations are decreased in insulin-resistant obese adults relative to equally obese insulin-sensitive controls. J Clin Endocrinol Metab. 2004;89(4):1630-5.

Meigs JB, Mittleman MA, Nathan DM, et al. Hyperinsulinemia, hyperglycemia, and

impaired hemostasis: the Framingham Offspring Study. Jama 2000; 283:221-8.

Melzack R. The McGill Pain Questionnaire: major properties and scoring methods.

Pain. 1975;1(3):277-99.

Mokdad, A.H., M.K. Serdula, et al. The spread of the obesity epidemic in the United States, 1991-1998. Jama. 1999; 282(16): 1519-22.

Monk TH, Buysse DJ, Welsh DK, Kennedy KS, Rose LR. A sleep diary and questionnaire study of naturally short sleepers. J Sleep Res. 2001;10(3):173-9.

Mustillo S, Worthman C, Erkanli A, Keeler G, Angold A, Costello EJ. Obesity and psychiatric disorder: developmental trajectories. Pediatrics, 2003; 111 (4 Pt 1): 851-9.

Palermo TM, Kiska R. Subjective sleep disturbances in adolescents with chronic pain: Relationship to daily functioning and quality of life. J Pain. 2005;6(3):201-7.

Pine DS, Cohen P, Brook J, Coplan JD. Psychiatric symptoms in adolescencee as predictors of obesity in early adulthood: a longitudinal study. American Journal of Public Health 87, 1303-1310.

Plasqui G, Westerterp KR. Seasonal variation in total energy expenditure and physical activity in Dutch young adults. Obes Res. 2004;12(4):688-94.

Pollak C, and Bright D. Caffeine consumption and weekly sleep patterns in US seventh-, eighth- and ninth-graders. Pediatrics. 2003; 111(1): 42-46.

Rosano C, Simonsick EM, Harris TB, Kritchevsky SB, Brach J, Visser M, Yaffe K, Newman AB. Association between physical and cognitive function in healthy elderly: the health, aging and body composition study. Neuroepidemiology. 2005;24(1-2):8-14.

Sleep hygiene. (n.d.) Retrieved March 14, 2005, from Universit;y of Maryland Sleep Disorders Center at http://www.umm.edu/sleep/sleep_hyg.html

Spiegel K, Tasali E, Penev P, Van Cauter E. Brief communication: sleep curtailment in healthy young men is associated with decreased leptin levels, elevated ghrelin levels, and increased hunger and appetite. Ann Intern Med. 2004; 141:846-50.

Spiegel K, Leproult R, Van Cauter E. Impact of sleep debt on metabolic and endocrine function. Lancet. 1999;354(9188):1435-9.

Taheri et al. A population-based study demonstrating that sleep loss is associated with increased body mass index, reduced leptin, and increased Ghrelin levels

OR19-6 Endocrine Society 2004.

Toppila J, Porkka-Heiskanen T. Transcriptional activity in the brain during sleep deprivation. Ann Med. 1999 Apr;31(2):146-51.

Van Cauter E, Leproult R, Plat L. Age-related changes in slow wave sleep and REM sleep and relationship with growth hormone and cortisol levels in healthy men. JAMA. 2000;284(7):861-8.

Van Cauter E, Spiegel K. Sleep as a mediator of the relationship between socioeconomic status and health: a hypothesis. Ann N Y Acad Sci. 1999;896:254-61.

Vorona R. Winn MP, Babineau TW, Eng BP, Feldman HR, Ware C Overweight and obese patients in a primary care population report less sleep than patients with a normal body mass index Arch Intern 2005; 165:25-30.

Webb WB and Agnew HW. Sleep stage characteristics of long and short sleepers

Science 1970; 18:146-147.

Wehr TA. Effect of seasonal changes in day length on human neuroendocrine function.

Horm Res. 1998;49(3-4):118-24.

Westbrook PR, Levendowski DJ, Cvtinovic M, Zavora T, Velimirovic V, Henninger D, Nicholosn D. Description and validation of the Apnea Risk Evaluation System: A novel method to diagnose sleep apnea-hypopnea in the home. Chest 2005; 128:2166-2175.

Wisse BE. The inflammatory syndrome: the role of adipose tissue cytokines in metabolic disorders linked to obesity. J Am Soc Nephrol. 2004;15:2792-800

Wolf AM, Colditz GA. Social and economic effects of body weight in the United States.

Am J Clin Nutr. 1996;63(3 Suppl):466S-469S.

Youngstedt SD, Kripke DF. Long sleep and mortality: rationale for sleep restriction. Sleep Med Rev. 2004 Jun;8(3):159-74.

**APPENDIX 1 Time Line of Study**

| EFFICACY PHASE  (Randomized Phase) | - 5 weeks | Screening Visit |
| --- | --- | --- |
|  | - 3 weeks | Randomization Visit (Inpatient) |
|  | 0 months | Baseline Assessment Visit (Inpatient) |
|  | + 1 month | Interim Visit |
|  | + 2 months | Interim Visit |
|  | + 4 months | Interim Visit |
|  | + 6 months | Interim Visit |
|  | + 8 months | Interim Visit |
|  | + 10 months | Interim Visit |
|  | + 12 months | End Point Assessment (Inpatient) |
| EFFECTIVENESS PHASE  (3 Year Follow-up Phase) | + 18 months | Follow-up Visit (Outpatient) |
|  | + 24 months | Follow-up Visit (Inpatient) |
|  | + 30 months | Follow-up Visit (Outpatient) |
|  | +36 months | Follow-up Visit (Inpatient) |
|  | +42 months | Follow-up Visit (Outpatient) |
|  | +48 months | Follow-up Visit (Inpatient) |

TABLE OF VISITS AND PROCEDURES

| **Study phase** | **Effiicacy Study (Randomized Phase)** | | | | | | | | | |
| --- | --- | --- | --- | --- | --- | --- | --- | --- | --- | --- |
| *Visit* | *Phone*  *screen* | | *Screening* | *Randomization/Baseline* | *0 Month* | *Month*  *1* | | *Month 2,4,6,8,10* | *Month 12* | |
| *When scheduled* | *-6w* | | *-5w* | *-3w* | *0 m* | *+1 m* | | *+2,4,6,8,10*  *m* | | *+12*  *m* |
| *Duration*  *(hrs)* | *1* | | *5* | *48*  *(2 n)* | *8* | *4* | | *4* | *48*  *(2 n)* | |
| ***General and sleep assessment*** |
| Phone interview | X | |  |  |  | |  |  |  | |
| Informed consent |  | | X | X |  | |  |  |  | |
| H & P |  | | X | X |  | |  |  | X | |
| Serum pregnancy |  | | X |  | X | |  |  | X | |
| Sleepiness scale |  | | X | X | X | | X | X | X | |
| PVT |  | |  | X | X | | X | X | X | |
| Apnea Screen (home) |  | | X |  |  | |  |  | X | |
| Activity watch |  | | X | X | X | | X | X | X | |
| MSLT |  | |  | X |  | |  |  | X | |
| Sleep diary |  | | X | X | X | | X | X | X | |
| PSQI Sleep questionnaire |  | | X | X | X | | X | X | X | |
| Physical activity questionnaire(AAFQ) |  | | X | X | X | | X | X | X | |
| Polysomnography |  | |  | X |  | |  |  | X | |
| Randomization |  | |  | X |  | |  |  |  | |
| Sleep instruction |  | |  | X |  | |  |  |  | |
| Health Education |  | |  |  |  | |  |  | X | |
| Compliance check |  | |  | X | X | | X | X | X | |
| ***Miscellaneous assessments*** |
| Quality of life  (SF-36, SIP, QLES) |  |  | | X |  | |  |  | X | |
| Sexual Satisfaction visual analog scale |  |  | | X |  | |  |  | X | |
| SCID |  |  | |  | X | |  |  |  | |
| Personality  (NEO, CookMedley, CIDI) |  |  | | X |  | |  |  |  | |
| Mood, anxiety ratings  (Hamilton, Beck) |  | | X | X | X | | X | X | X | |
| 3-day food record |  |  | | X |  | |  | X(4) | X | |
| 24 hour food recall |  |  | |  | X | | X | X | X | |
| Appetite (Visual Analog Scales) |  |  | | X | X | | X | X | X | |
| Activity monitor (two weeks of month) |  |  | | X | X | | X | X | X | |
| Cooper or step test |  |  | | X |  | |  |  | X | |
| Indirect calorimetry |  |  | | X |  | |  |  | X | |
| Screening for chronic pain syndromes |  |  | | X |  | |  |  | X | |
| Neuropsych testing |  |  | | X |  | |  |  | X | |
| Unicorder |  | X | |  |  | |  |  | X | |
| *Menstrual Calendar |  | X | | X | X | | X | X | X | |

| *Visit* | *Phone*  *screen* | *Screening* | *Randomization/Baseline* | *0 Month* | *Month*  *1* | *Month 2,4,6,8,10* | | *Month 12* |
| --- | --- | --- | --- | --- | --- | --- | --- | --- |
| *When scheduled* | *-6w* | *-5w* | *-3w* | *0 m* | *+1 m* | *+2,4,6,8,10*  *m* | | *+12*  *m* |
| *Duration*  *(hrs)* | *1* | *5* | *48*  *(2 n)* | *8* | *4* | *4* | | *48*  *(2 n)* |
| ***Assessment for Metabolic Syndrome*** |
| Height and weight |  | X | X | X | X | X | X | |
| Resting pulse and BP  Temperature |  | X | X | X | X | X | X | |
| Waist, hip and neck circumference |  | X | X | X | X | X | X | |
| Fasting insulin and glucose |  | X | X | X | X | X | X | |
| Hgb A1c |  | X | X | X | X | X | X | |
| Fasting lipid panels |  | X | X | X | X | X | X | |
| Oral GTT |  |  | X |  |  |  | X | |
| Plethysmography  (BOD POD) |  |  |  | X |  |  | X | |
| DEXA body composition |  |  |  | X |  |  | X | |
| Bioelectric impedance |  |  |  | X |  |  | X | |
| CT abdominal fat |  |  |  | X |  |  | X | |
| ***Endocrine and Immume Evaluation*** |
| 8am Leptin, ghrelin, & adiponectin |  |  | X | X | X | X | X | |
| 8am plasma ACTH and cortisol |  |  | X |  |  |  | X | |
| UFC |  |  | X |  |  |  | X | |
| 8am FT4 and TSH |  |  | X |  |  |  | X | |
| 8am GH IGF-1 |  |  | X |  |  |  | X | |
| Total and Free T |  |  | X |  |  |  | X | |
| *Estradiol |  | X | X | X | X | X | X | |
| *Progesterone |  | X | X | X | X | X | X | |
| *FSH & LH |  | X | X | X | X | X | X | |
| Inflammatory markers |  |  | X |  |  |  | X | |

*Women only

Table of Visits and Procedures

| **Study Phase** | **Effectiveness Study (3 Year Follow-up Phase)** | | | | | |
| --- | --- | --- | --- | --- | --- | --- |
| *Effectiveness Visit*  *(3 Year Follow-up)* | *Month 6*  *Interim Visit* | ***Month 12***  ***Main Visit*** | *Month*  *18*  *Interim Visit* | ***Month***  ***24***  ***Main Visit*** | *Month*  *30*  *Interim Visit* | ***Month***  ***36***  ***Main Visit*** |
| *Duration* | *6 hrs* | **Screening 5 hrs*  *1-3 n* | *6 hrs* | **Screening 5 hrs*  *1-3 n* | *6 hrs* | **Screening 5 hrs*  *1-3 n* |
| ***General and sleep assessment*** |
| Phone screening interview |  | X* |  | X* |  | X* | |
| Informed consent |  | X* |  | X* |  | X* | |
| H & P |  | X* |  | X* |  | X* | |
| Screening labs |  | X* |  | X* |  | X* | |
| EKG |  | X* |  | X* |  | X* | |
| Serum pregnancy |  | X |  | X |  | X | |
| Sleepiness scale | X | X | X | X | X | X | |
| Apnea Screen (Unicorder) |  | X |  | X |  | X | |
| Activity watch | X | X | X | X | X | X | |
| MSLT |  | X |  | X |  | X | |
| Sleep diary | X | X | X | X | X | X | |
| Epworth Sleepiness and PSQI Sleep questionnaire | X | X | X | X | X | X | |
| Reaction time testing (PVT) | X | X | X | X | X | X | |
| Polysomnography |  | X |  | X |  | X | |
| Review of Sleep instruction (only for original subjects) | X | X | X | X | X | X | |
| Health Education (Diet and Exercise) (original subjects) | X | X |  | X |  | X | |
| Compliance check | X | X | X | X | X | X | |

*X =for External Comparisons only

| *Effectiveness Visit*  *(3 Year Follow-up Phase)* | *Month 6*  *Interim Visit* | ***Month 12***  ***Main Visit*** | *Month*  *18*  *Interim Visit* | ***Month***  ***24***  ***Main Visit*** | *Month*  *30*  *Interim Visit* | ***Month***  ***36***  ***Main Visit*** |
| --- | --- | --- | --- | --- | --- | --- |
| *Duration* | *6 hrs* | **Screening 5 hrs*  *1-3 n* | *6 hrs* | **Screening 5 hrs*  *1-3 n* | *6 hrs* | **Screening 5 hrs*  *1-3 n* |

| ***Miscellaneous assessments*** |
| --- |
| Quality of life  (SF-36, SIP,) |  | X |  | X |  | X |
| Sexual  Satisfaction Visual Analogue |  | X |  | X |  | X |
| Pain Assessment |  | X |  | X |  | X |
| SCID |  | X |  | X |  | X |
| Mood, anxiety rating  (Hamilton Scales for Depression and anxiety) | X | X | X | X | X | X |
| 3-day food record |  | X |  | X |  | X | |
| Appetite (Visual Analog Scales) | X | X | X | X | X | X | |
| Actical activity monitor and pedometer | X | X | X | X | X | X | |
| Physical activity questionnaire (AAFQ) | X | X | X | X | X | X | |
| Indirect calorimetry |  | X |  | X |  | X | |
| Neuropsychological testing |  | X |  | X |  | X | |
| Weight recording self-report | X | X | X | X | X | X | |
| Blood Pressure recording self-report | X | X | X | X | X | X | |
| Menstrual calendar self-report (women) | X | X | X | X | X | X | |

*X= for External Comparisons only

| *Effectiveness Visit*  *(3 Year Follow-up Phase)* | *Month 6*  *Interim Visit* | ***Month***  ***12***  ***Main Visit*** | | *Month*  *18*  *Interim Visit* | ***Month***  ***24***  ***Main Visit*** | *Month*  *30*  *Interim Visit* | ***Month***  ***36***  ***Main Visit*** |
| --- | --- | --- | --- | --- | --- | --- | --- |
| *Duration* | *6 hrs* | **Screening 5 hrs*  *Stay 1-3 n* | | *6 hrs* | **Screening 5 hrs*  *Stay 1-3 n* | *6 hrs* | **Screening 5 hrs*  *Stay 1-3 n* |
| ***Assessment for Metabolic Syndrome*** |
| Height* and weight | X | | X | X | X | X | X |
| Resting pulse and BP  Temperature | X | | X | X | X | X | X |
| Waist and neck circumference | X | | X | X | X | X | X |
| Fasting insulin and glucose | X | | X | X | X | X | X |
| Hgb A1c | X | | X | X | X | X | X |
| Fasting lipid panels | X | | X | X | X | X | X |
| Oral GTT |  | | X |  | X |  | X |
| Plethysmography  (BOD POD) |  | | X |  | X |  | X |
| DEXA body composition |  | | X |  | X |  | X |
| Bioelectric impedance |  | | X |  | X |  | X |
| CT abdominal fat |  | | X |  | X |  | X |
| ***Endocrine and Immune Evaluation*** |
| 8am Leptin, ghrelin, & adiponectin | X | X | | X | X | X | X |
| 8am plasma ACTH and cortisol |  | X | |  | X |  | X |
| 24h urinary free cortisol |  | X | |  | X |  | X |
| 8am FT4 and TSH |  | X | |  | X |  | X |
| 8am GH IGF-1 |  | X | |  | X |  | X |
| Total and Free T |  | X | |  | X |  | X |
| Estradiol (women) | X | X | | X | X | X | X |
| Progesterone (women) | X | X | | X | X | X | X |
| FSH & LH (women) | X | X | | X | X | X | X |
| Inflammatory markers |  | X | |  | X |  | X |

X*= External Comparisons only

# APPENDIX 2

**Description of Procedures**

Activity Watch:

This instrument is routinely used in the study of sleep in large populations. The instrument will be placed on the non-dominant wrist of the subject, and data will be downloaded to a computer at the NIH CC when the subject comes back. The actigraphy will provide a complement to the sleep diaries. The subject will maintain an activity log and will also record when the watch is put on or taken off for brief periods of time for showering or similar reasons. We will ask subjects to wear the activity watch for two weeks at a time prior to each visit, except initially when it will be worn from the screening to the randomization visit (approximately 2 weeks). The instrument is referred to as the Actiwatch.

Activity Monitor:

This instrument is very similar to the activity watch in principle of operation and in appearance. It is worn at the waist on an elastic belt, or clipped to the waistband or belt. We asked subjects to wear it except when sleeping or bathing, for two weeks prior to each visit after the Randomization. From the downloaded data, activity can be analyzed to determine the time spent in varying levels of activity, and the amount of energy (calories) expended in activity. It is referred to as the Actical.

BOD POD (plethysmography):

The BOD POD, a dual-chambered (test chamber and reference chamber) fiberglass plethysmograph determines body volume by measuring changes in pressure within a closed chamber. The subject sits inside a comfortable chamber, and the volume of air displaced by the subject’s body determines body composition. From mass and volume whole-body density can be determined, and body fat and lean mass calculated.

Bioelectric Impedance Analysis (BIA)

A handheld device is used to calculate body fat. An unfelt, safe, extremely low energy, high frequency electrical signal is sent from one contact point on the body to another, and a measurement of baseline impedance to the flow of the signal is made as it travels through the body. Based on the conductivity of fat tissue and water, the device calculates the speed at which the electrical signal travels through the body’s tissues and the analyzer uses the electrical resistance information to calculate body fat weight and body fat percentage.

Cooper Test:

The Cooper test is a test of aerobic capacity and physical fitness that has been widely validated in different age ranges and patient populations, and is highly predictive of the VO2 max with a coefficient of correlation of 0.87. Subjects will be asked to cover as much distance as possible in 12 minutes by running and/or walking, with an 8-minute recovery period at the end where the subject walks at his/her own pace. The total distance covered in 12 minutes will be considered the index of endurance. The heart rate is continuously monitored by a telemeter during exercise and recovery for safety reasons, and may be recorded to document compliance with maximum effort. Heart rate/velocity ratio serves as an indirect index of endurance.

(GTT) Oral glucose tolerance test:

Subjects will be instructed to eat meals containing at least 200 grams of carbohydrates a day for 3 days before testing. The test will be performed starting between 8 am and 10 am as reported. A 75-gram glucose load is administered: blood is drawn at 0, 30, 60, 90, and 120 minutes. Insulin and glucose are measured.

MSLT (multiple sleep latency test):

The MSLT is a series of four to six nap opportunities in a dark and quiet room presented at two-hour intervals beginning approximately two hours after initial (morning) awakening. The subjects will be instructed to allow themselves to fall asleep or not to resist falling asleep. Subjects are not permitted to remain in bed between nap test sessions. Electrophysiological parameters to detect sleep onset and score sleep stages are recorded during nap opportunities (central and occipital EEGs, left and right eye EOGs, and submentalis EMGs). Respiratory flow and sounds may also be monitored.

PSG (polysomnography):

PSG, monitoring and recording physiologic data during sleep, is done to evaluate sleep-related respiratory disturbances and provide information on sleep stages. The measures may include global neural electroencephalographic activity (EEG) from electrodes placed on the subject’s scalp, eye movements (electrooculogram or EOG) from electrodes placed near the outer canthus of each eye, submental electromyographic activity (EMG) from electrodes placed over the mentalis, rhythm ECG with two or three chest leads, respiratory effort by strain gauge or intercostal EMG, nasal and/or oral airflow by thermistor, oxygen saturation by pulse oximetry, and limb movements by EMG. The output of devices attached to the patient is interfaced with a polysomnographic recorder and displayed on a computer monitor. The testing takes place in a sound and light insulated room and the subject is monitored throughout the procedure. The EEG sleep stages will be compared with inpatient wrist activity levels in order to more reliably estimate sleep in the outpatient setting based on wrist activity data alone.

Psychomotor Vigilance Task (PVT):

The device measures reaction time, as an indicator of sleepiness or alertness. During a 10-minute test session, the subject holds the book-size instrument in her hands, and as lighted numbers count down in milliseconds, she presses a button to stop the count as quickly as she can, recording the time it takes to respond.

Unicorder (Apnea Risk Evaluation System [ARES]

The ARES Unicorder is a miniaturized monitor capable of recording oxygen saturation, pulse rate, snoring level, head position/movement and optional nasal pressure airflow. The ARES Unicorder is placed on the forehead by the user and comfortably worn for 8-10 hours. It is held in place by an elastic strap, and airflow is detected by a nasal cannula. Subjects will be instructed in the use of the device and take it home to obtain the recording for one night. The recording will be analyzed to quantify abnormal respiratory events. The combination of physiological data, questionnaire responses, and expert pattern recognition software provides an accurate and valid assessment of sleep-disordered breathing. It will be used to screen subjects for obstructive sleep apnea upon study entry.

YMCA 3-Minute Step Test:

This test is used to evaluate fitness during submaximal efforts. The subject steps up and down on a 12-inch platform at a rate of 24 steps per minute for 3 minutes, and then sits down. The tester takes the subject’s heart rate for one full minute, which is the test score.

**Glossary of Terms**

Actigraph

When the data from the activity watch is read out, an actigraph showing amount of physical movement for an interval of time is generated.

Activity log

An activity log will be used with the activity watch and monitor. The time that the activity watch is put on is recorded, and the subject will record the time they take it off and put it back on when showering or performing other activities where the watch cannot be worn.

Exercise diary

Subjects will record their physical activity on an exercise log. Such activity will include recreational exercise but also daily activities and household chores that require physical exertion.

Food records

Records of food intake, which the subjects will keep, will include a food diary, which will be filled out for three days at the beginning, 4-month visit, and end of the Efficacy (Randomized Phase) study.

Sleep diary

Subjects will record their sleep time, including any naps, on a record. This diary will be completed for the two weeks preceding the randomization visit, and for two weeks preceding each of the subsequent visits. It will be employed to measure sleep duration upon study entry, sleep duration during the run-in period following randomization to intervention or comparison group, and sleep duration during the remainder of the study. Information from the sleep diary will be compared to the actigraph generated from the activity watch data, which will serve as a corroboration of the subject’s ability to monitor and accurately record sleep time.

**APPENDIX 3**

**Qualifications of Investigators**

**Giovanni Cizza**, **M.D., Ph.D.,** **MHSc**. is a Principal Investigator in the Clinical Endocrine Section, Clinical Endocrinology Branch, NIDDK, and is responsible for the study design and implementation of the Sleep Intervention Trial. He is also currently finalizing a protocol on energy expenditure in subjects with narcolepsy. He received training in adult endocrinology at Pisa University School of Medicine and at the NIH Interinstitute Program, Bethesda, MD. His Ph.D. in Clinical Pharmacology is from the Mario Negri Institute for Pharmacological Research, in Milano, Italy. Dr. Cizza recently obtained a Master of Health Sciences in Clinical Research from the Duke University, NC. He has several years of experience in clinical research on the obesity of Cushing’s syndrome, major depression, osteoporosis and on the clinical development of pharmacological entities such as alendronate and antalarmin, a novel CRH type 1 selective antagonist. He also had additional years of basic research focusing on the differential brain expression of neuropeptides in aged organisms during stress and other aversive conditions.

**Amber Courville, PhD, RD** is a Metabolic Research Dietitian for the Clinical Nutrition Department at the NIH Clinical Center. She received her BS in Kinesiology and her PhD in Nutrition from the University of Connecticut. Amber completed her dietetic internship at NIH. Before returning to NIH in January of 2009, Amber worked at the USDA Beltsville Human Nutrition Research Center conducting clinical research studies examining the effects of nutrients on metabolism and disease. As a metabolic research dietitian for the NIH Intramural Obesity Research Initiative, Amber is responsible for the design, calculation and implementation of metabolic diets required for obesity-related studies as well as for conducting research related to the study of metabolism and obesity.

**Elizabeth (Lilian) de Jonge-Levitan, MSc, Ph.D**. obtained her undergraduate and MSC degrees in Nutrition from the Agricultural University in Wageningen, The Netherlands, and her PhD in Nutrition from the Université de Montréal in, Montréal, Canada. After her PhD, she moved to the Pennington Biomedical Research Center in Baton Rouge, LA in 1996 for post-doctoral training. She became a faculty member at the Pennington Biomedical Research Center after one year of post-doctoral training and served as the director of the metabolic chambers laboratory until August 2010. Dr. de Jonge is employed by Kelly Government Solutions as a contractor to work with the Clinical Endocrinology Branch of NIDDK.

**Wallace C. Duncan Jr., Ph.D**. is Research Psychologist who is actively engaged in clinical research in the Mood and Anxiety Disorders Program (MAP) at the National Institutes of Mental Health in Bethesda, Maryland. Dr. Duncan will be responsible for assessment of sleep durations based on the use of polysomnogram, actiwatch and sleep diaries during the protocol. He has extensive training and research experience in sleep and waking processes, as well as circadian and seasonal rhythms; he is the principal investigator of a research protocol investigating the treatment of winter depression by early morning inhibition of melatonin secretion using beta-blockers. His Ph.D is in Biological Sciences from the University of Maryland and he has extensive experience with the use of actigraphy as a biological marker of sleep and the circadian clock. In the MAP program he is actively engaged in a research program that is investigating the relationship between neural plasticity, sleep and mood disorders.

**Gregor Hasler, M.D**., is a psychiatrist and psychiatric epidemiologist trained at Zurich University (supervisor: Dr. Jules Angst). He conducted several studies on the behavioral correlates, including sleep duration, of body weight and obesity using data from the Zurich Cohort study, a large community study with a follow-up over 20 years. Besides his work on weight problems, he is focusing on problems related to the diagnostic classification in psychiatry and the integration of environmental, neurobiological, and genetic information into a new classification system based on etiology and pathophysiology. Dr. Hasler is currently working as a senior scientist at the Department of Psychiatry, University Hospital, Zurich, Switzerland. Among others, he is conducting a study on the role of catecholamines in the pathophysiology of eating disorders.

**Megan Mattingly, RN, MPH**, is a research nurse specialist and is the study coordinator. She received her Bachelors of Science in Nursing from the University of Vermont and her Masters of Public Health from Johns Hopkins Bloomberg School of Public Health. Prior to joining the team at NIDDK, Ms. Mattingly served as a nurse in the U.S. Navy for 11 years and worked for one year as a clinical research nurse at the NIH clinical center.

**Nancy Sebring, MEd, RD**, has been a Clinical Research Dietitian at the NIH Clinical Center for the past 20+ years. She received her BS in Nutrition from the University of Delaware, completed a dietetic internship at Emory University (Atlanta, GA), and received her Masters Degree in Education from the University of Maryland. She obtained a Certificate in Training in Adult Weight Management from the American Dietetic Association in 2003. She coordinates and performs assessment of dietary assessment and body composition for a variety of clinical studies, and provides clinical nutrition services for endocrinology patients at the Clinical Center.

**Monica C. Skarulis, M.D.** is the chief of the Clinical Endocrine Section, Clinical Endocrinology Branch, NIDDK and the Director of the NIH Inter-Institute Fellowship Program in Endocrinology, Diabetes and Metabolism. In addition to her studies in endocrine neoplasia which include the trials of recombinant human thyrotropin in thyroid cancer, she is currently leading the development of the new Metabolic Research Unit in the new Hatfield Clinical Research Center. This new initiative includes an 11 bed inpatient unit, invasive metabolic testing area, three respiratory chambers for indirect calorimetry, and body composition, exercise, and eating behavior laboratories.

Qualifications of Collaborators

**David Allison Ph.D.** is a biostatistician who is currently Distinguished Professor and Associate Dean for Science at the University of Alabama at Birmingham and the Director of the NIH-funded Nutrition Obesity Research Center. He has authored over 450 scientific publications, edited five books and has received multiple awards for his work. Dr. Allison’s research interests include obesity, quantitative genetics, clinical trials, and statistical and research methodology.

**Dr. Richard Atkinson, M.D**. is a clinical Professor of Pathology and Virginia Commonwealth University and the Director of Obetech Obesity Research Center. He has published over 170 manuscripts and over 200 abstracts in the medical literature. Recently his research has focused on virus-induced obesity.

**Nikhil Dhurandhar, Ph.D.,** is a professor in the Department of Infections and Obesity at the Pennington Biomedical Research Center in Baton Rouge, LA. His lab is currently researching obesity of infectious origin. The long term goal of his lab is to discover treatment and/or prevention of Ad-36 induced adiposity.

**APPENDIX 4**

Compensation of subjects

**Schedule of payments**

Subjects will be compensated in accordance with the guidelines of the NIH Volunteer Office. The guidelines prescribe payment amounts for overnight stays, outpatient visits by the time involved, and additionally, the assignment of Inconvenience Units for procedures.

Overnight stays $40/night

Outpatient visits $20 for first hour, $ 10 for subsequent hours

Inconvenience units $10 for each unit

**Assignment of Inconvenience Units**

DEXA scan 6

CT scan 6

PSG 5

Exercise (Cooper or Step) 3

Oral GTT 5 (includes blood draw and fasting)

Indirect calorimetry 1

Bod Pod 2

Bioelectric impedance 1

Phlebotomy (blood draw) 2

Fasting 1

24 hour urine collection 2

Actiwatch 2 (for 1 week)

MSLT 3

Neuropsych testing 2

H and P 0

Questionnaires

Sleep 0

QOL 0

Personality 0

Sleep Diaries 2 (for 1 week)

Exercise Diaries, Actical 2 (for 1 week)

Food Diaries 2 (for 3 days)

Scales (Appetite, Sleepiness) 0

Ratings (Mood, Anxiety) 0

Weight, BP, and Pedometer

Records 0

Measurements (waist, hip,

neck) 0

Pain Screening 0

SCID 0

**Compensation by visits**

Screening

4 hours $50

Fasting blood draw (3)

Activity Watch (4)

Sleep Diaries (4)

11 Inconvenience Units $110

$160

Randomization

3 nights $120

Activity watch and monitor (8)

Sleep, Exercise, & Food diaries (10)

PSG (5)

Fasting blood draw (3)

26 Inconvenience Units $260

$380

Baseline

1 night $40

Activity watch and monitor (8)

Sleep and Exercise diaries (4)

MSLT (3)

Exercise test (3)

Indirect calorimetry (1)

Neuropsych testing (2)

Oral GTT (5)

BOD POD (2)

BIA (1)

Fasting blood draw (3)

DEXA (4)

CT (8)

24-hour urine (2)

46 Inconvenience Units $460

$500

Months 1, 2, 4, 6, 8, 10

4 hours $50

Activity watch and monitor (8)

Sleep and Exercise diaries (4)

Fasting Blood Draw (3)

15 Inconvenience Units $150

$200 for each of 6 visits = $1200

Month 12 end-point

(same as Baseline with addition of PSG)

2 nights $80

51 Inconvenience Units $510

$590

Month 18 follow-up

2 nights $80

Fasting Blood Draw (3)

BIA (1)

MSLT (3)

PSG (5)

Sleep and Exercise Diaries (24)

36 Inconvenience Units $360

$340

TOTAL FOR ALL VISITS $3100

Months 18, 30, and 42 outpatient extension visits

6 hours $70

Fasting Blood Draw (3)

Activity Watch (2/week for 6 weeks)

Activity Monitor (2/week for 6 weeks)

Sleep Diaries (2/week for 6 weeks)

36 Inconvenience Units $390

$460

Months 24, 36, and 48 Inpatient Extension Visits (for originally enrolled subjects)

2 nights $80

OGTT (5)

Indirect Calorimetry (1)

Food Records (2)

BIA (1)

DEXA (6)

CT (6)

24-Hour Urine Collection (2)

Neuropsychological Testing (2)

Activity Watch (2/week for 6 weeks)

Activity Monitor (2/week for 6 weeks)

Sleep Diaries (2/week for 6 weeks)

61 Inconvenience Units $610

$690

Months 24, 36, and 48 inpatient Extension Visits (for external comparison subjects)

2 nights $80

OGTT (5)

Indirect Calorimetry (1)

Food Records (2)

BIA (1)

DEXA (6)

CT (6)

24-Hour Urine Collection (2)

Neuropsychological Testing (2)

25 Inconvenience Units $250

$330
